# Humanization reveals pervasive incompatibility of yeast and human kinetochore components

**DOI:** 10.1093/g3journal/jkad260

**Published:** 2023-11-14

**Authors:** Guðjón Ólafsson, Max A B Haase, Jef D Boeke

**Affiliations:** Institute for Systems Genetics and Department of Biochemistry and Molecular Pharmacology, NYU Langone Health, New York, NY 10016, USA; Institute for Systems Genetics and Department of Biochemistry and Molecular Pharmacology, NYU Langone Health, New York, NY 10016, USA; Vilcek Institute of Graduate Biomedical Sciences, NYU School of Medicine, New York, NY 10016, USA; Institute for Systems Genetics and Department of Biochemistry and Molecular Pharmacology, NYU Langone Health, New York, NY 10016, USA; Department of Biomedical Engineering, NYU Tandon School of Engineering, Brooklyn, NY 14 11201, USA

**Keywords:** humanization, yeast, kinetochore, centromere, histone variant, CENP-A

## Abstract

Kinetochores assemble on centromeres to drive chromosome segregation in eukaryotic cells. Humans and budding yeast share most of the structural subunits of the kinetochore, whereas protein sequences have diverged considerably. The conserved centromeric histone H3 variant, CenH3 (CENP-A in humans and Cse4 in budding yeast), marks the site for kinetochore assembly in most species. A previous effort to complement Cse4 in yeast with human CENP-A was unsuccessful; however, co-complementation with the human core nucleosome was not attempted. Previously, our lab successfully humanized the core nucleosome in yeast; however, this severely affected cellular growth. We hypothesized that yeast Cse4 is incompatible with humanized nucleosomes and that the kinetochore represented a limiting factor for efficient histone humanization. Thus, we argued that including the human CENP-A or a Cse4–CENP-A chimera might improve histone humanization and facilitate kinetochore function in humanized yeast. The opposite was true: CENP-A expression reduced histone humanization efficiency, was toxic to yeast, and disrupted cell cycle progression and kinetochore function in wild-type (WT) cells. Suppressors of CENP-A toxicity included gene deletions of subunits of 3 conserved chromatin remodeling complexes, highlighting their role in CenH3 chromatin positioning. Finally, we attempted to complement the subunits of the NDC80 kinetochore complex, individually and in combination, without success, in contrast to a previous study indicating complementation by the human *NDC80/HEC1* gene. Our results suggest that limited protein sequence similarity between yeast and human components in this very complex structure leads to failure of complementation.

## Teaser

Ólafsson, Haase, and Boeke test whether the human histone H3 centromere-specific variant, CENP-A, and the key outer kinetochore complex, NDC80/HEC1, can complement for their yeast counterparts. The authors hypothesized that human CENP-A would alleviate a centromere/kinetochore phenotype in histone-humanized yeast but found that the genes encoding human CENP-A and the NDC80 complex cannot replace their yeast equivalents. In fact, CENP-A overexpression is lethal in wild-type yeast. Their results highlight the importance of species specific chromatin remodeling for centromeric function.

## Introduction

**Fig. 1.**
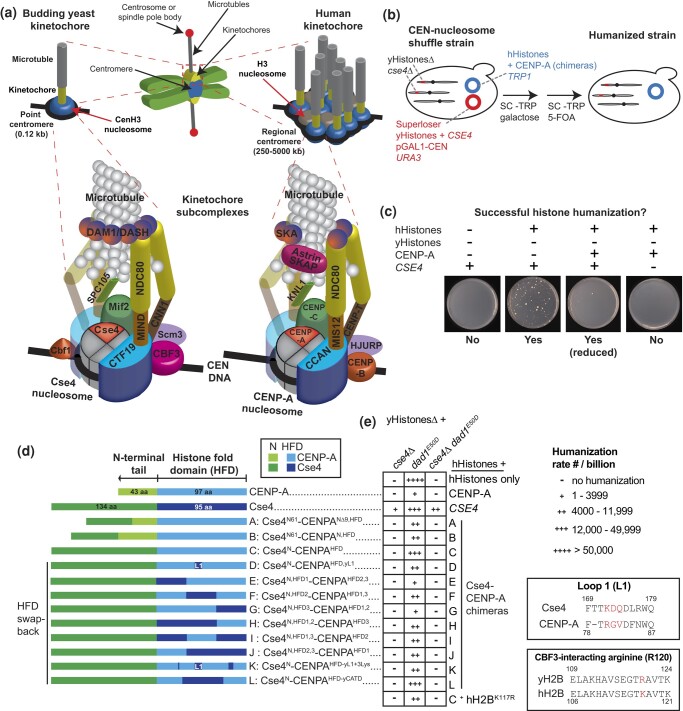
Humanization of the Cse4 nucleosome with the CENP-A nucleosome in yeast. a) Illustration of the centromere and kinetochore in budding yeast and human cells. The kinetochore cartoon highlights the similarities and differences between yeast and human kinetochores and their subcomplexes. The cartoon is not to scale and is not meant to signify an actual kinetochore structure. The kinetochore cartoon is based on a previous model of the kinetochore architecture (Biggins 2013) and is adapted from Klemm et al. (2020) and Ólafsson and Thorpe (2020). b) Overview of the Superloser dual-plasmid shuffle assay (see Supplementary Fig. 1a for schematics of the Superloser and CEN nucleosome humanization plasmids and methods for details). c) Example images of agar plates from the histone humanization assay of *yHsΔ dad1^E50D^ CSE4+* shuffle strain showing histone-humanized colonies after 20 days of growth at 30°C on SC–Trp 5-FOA media. For histone humanization experiments, we use a positive control: a plasmid containing hHs and yeast *CSE4* (instead of CENP-A) and 2 negative controls: 1 containing hHs only and the other an empty plasmid to control for recombination. As an additional control, we also performed our experiments using the *dad1^E50D^* histone shuffle strain containing endogenous *CSE4* (*CSE4*+). Note that the few large colonies that typically appear within 3 days represent plasmid recombinants or spontaneous *ura3* mutants that have gained 5-FOA resistance (see main text for details). d) Schematics of the various Cse4–CENP-A chimeras are shown and are in line with the data in panel e). e) The histone humanization rate data are summarized in a table format (see bar graph of the same data in Supplementary Fig. 1d and the raw data in Supplementary Table 3). Insert (top) displays aa sequence alignment of the loop 1 of Cse4 and CENP-A, and the nonconserved three amino acid sequence region is highlighted. Insert (bottom) shows the alignment of the region of yH2B and hH2B. The nonconserved arginine residue that is important for interaction with the CBF3 complex is highlighted (see main text for details).

To ensure accurate chromosome segregation during cell division, chromosomal regions termed centromeres assemble a large multiprotein structure known as the kinetochore (Fig. 1a; Biggins 2013). Kinetochores attach to microtubules emanating from the poles, or centrosomes in metazoans and spindle pole bodies in yeasts, thus connecting and orienting sister chromatids on the mitotic spindle, allowing segregation into daughter cells. Centromeres contain a specialized histone H3 variant CenH3, CENP-A in humans and Cse4 in *Saccharomyces cerevisiae*, which replaces canonical histone H3 at centromeres. The relatively simple point centromere of *S. cerevisiae* is genetically defined by a ∼120 base-pair (bp) DNA sequence that wraps around a single Cse4 nucleosome, which assembles a single kinetochore that can bind 1 microtubule. The point centromere is subdivided into 3 DNA elements (CDEI, CDEII, and CDEIII): CDEII is a 78–86 bp sequence rich in adenine and thymine (AT) and flanked by 2 conserved sequences, the 8-bp CDEI and the 26-bp CDEIII, which bind the nonessential transcription factor Cbf1 and the essential budding yeast-specific CBF3 complex, respectively.

Kinetochores regulate microtubule attachment to ensure high-fidelity segregation of chromosomes. Disruption of this process causes chromosomal instability and aneuploidy, which are hallmarks of cancer cells. For example, CENP-A overexpression is commonly observed in cancer cells which results in its mislocalization to euchromatin, causing ectopic neocentromere formation and genomic instability (Lacoste *et al*. 2014; Athwal *et al*. 2015; Chung *et al*. 2016; Shrestha *et al*. 2017, 2021; Nye *et al*. 2018; Sharma *et al*. 2019; Mahlke and Nechemia-Arbely 2020). Ubiquitin-mediated proteolysis of CenH3 is a conserved mechanism that limits excessive CenH3 levels and its misincorporation into noncentromeric chromatin (Dong *et al*. 2021). In budding yeast, *CSE4* overexpression is only toxic to cells lacking Cse4 regulators such as E3 ubiquitin ligase Psh1 (Collins *et al*. 2004; Hewawasam *et al*. 2010; Ranjitkar *et al*. 2010; Cheng *et al*. 2017). In yeast and humans, CenH3 misincorporation disrupts global transcriptional regulation and chromosome segregation (Hildebrand and Biggins 2016; Hewawasam *et al*. 2018; Dong *et al*. 2021).

The point centromeres of budding yeasts are sometimes incorrectly presumed to be evolutionary precursor of more complex regional centromeres of metazoans. However, evidence suggests that the budding yeast lineage once contained regional centromeres, which gradually minimized over an evolutionary timescale (Dalal *et al*. 2007; Malik and Henikoff 2009; Lefrançois *et al*. 2013; Talbert and Henikoff 2020). Human regional centromeres are epigenetically defined and can span several megabases of repetitive DNA sequence arrays. The sites of kinetochore assembly are within smaller subregions ranging from 190 to 570 kilobases and contain a relatively high CENP-A nucleosome enrichment interspersed with canonical H3 nucleosomes (Altemose *et al*. 2022). CENP-A nucleosomes are wrapped by repetitive ∼170 bp AT-rich alpha satellite sequences. Thus, many kinetochores assemble on a single human centromere supporting multiple microtubule attachments.

In contrast to centromeres, kinetochore architecture is relatively well conserved, despite approximately a billion years of divergent evolution (Kitagawa and Hieter 2001), and shares most of the key structural elements and the overall kinetochore “layout” (Fig. 1a; Burrack and Berman 2012; Westermann and Schleiffer 2013; Musacchio and Desai 2017; Hamilton *et al*. 2019). In both species, the inner kinetochore binds the CenH3 nucleosome and consists of CENP-C^Mif2^ and a 15–16 subunit complex—the constitutive centromeric-associated network (CCAN)—bridging the centromere and outer kinetochore that attaches to microtubules. The outer kinetochore in both species shares 3 key subcomplexes, collectively termed the KMN network (KNL1^SPC105^, MIS12^MIND^, and NDC80). The NDC80 complex (NDC80c) provides the major microtubule-binding interface. In *S. cerevisiae*, NDC80c interacts with the yeast-specific DAM1/DASH complex, which forms a ring-like structure around the microtubule and is essential for kinetochore function; the nonhomologous human SKA complex is proposed to be its functional counterpart (Musacchio and Desai 2017; van Hooff *et al*. 2017).

Due to similarities between *S. cerevisiae* and human kinetochores, yeast has proven to be an invaluable model for understanding human kinetochore structure and function (Yamagishi *et al*. 2014). This has also prompted the notion that the human kinetochore is effectively composed of repetitive arrays of “kinetochore-nucleosome units,” comparable with a single Cse4 nucleosome–based *S. cerevisiae* kinetochore (Fig. 1a). Correspondingly, the cluster of 16 centromeres/kinetochores forms the analogous entity as a single human centromere/kinetochore (Fig. 1a; Zinkowski *et al*. 1991; Bouck *et al*. 2008; Bloom 2014; Pesenti *et al*. 2016; Weir *et al*. 2016; McAinsh and Marston 2022). However, this idea has not been directly tested nor has the extent to which yeast and human kinetochores are comparable in vivo been systematically evaluated.

Complementation studies that individually replace yeast genes with human orthologs have been used for decades and increased our understanding of fundamental cellular processes important for human health (Beach *et al*. 1982; Lee and Erikson 1997; Li *et al*. 1997; Zheng *et al*. 2000; Dotan *et al*. 2001; Zhang *et al*. 2003; Mager and Winderickx 2005; Franssens *et al*. 2013; Hamza *et al*. 2015, 2020; Laurent *et al*. 2016; Yang *et al*. 2017; Lubrano *et al*. 2019; Kim *et al*. 2020; Roohvand *et al*. 2020; Kachroo *et al*. 2022). However, only few studies have attempted to replace kinetochore genes (Chang *et al*. 1996; Kitagawa *et al*. 1999; Kitagawa and Hieter 2001). Stoler *et al*. discussed but did not show that human CENP-A could not complement Cse4 in *S. cerevisiae* (Stoler *et al*. 1995). However, co-complementation with core histones was not attempted. Strikingly, it was reported that the human Ndc80 subunit (also known as “highly expressed in cancer 1” HEC1) can fully complement its ortholog in *S. cerevisiae* (Zheng *et al*. 1999), despite a <30% amino acid (aa) sequence identity. However, whether other NDC80c subunits, Nuf2, Spc24, and Spc25, are replaceable is undetermined.

More recently, yeast humanization of whole pathways and protein complexes with human counterparts in yeast has been achieved (Huber *et al*. 2016; Laurent *et al*. 2016; Truong and Boeke 2017; Agmon *et al*. 2019; Garge *et al*. 2020; Kim *et al*. 2020; Roohvand *et al*. 2020; Boonekamp *et al*. 2022; Kachroo *et al*. 2022; Abdullah *et al*. 2023; Sultana *et al*. 2023). For example, our lab successfully humanized the nucleosome in *S. cerevisiae* by swapping out the yeast core histone (yHs) variants for their human counterparts, H3, H4, H2A, and H2B (Truong and Boeke 2017). However, the histone humanization frequency was very low, and the resulting strains showed extremely slow growth, aneuploidy, and rapid adaptation by evolving suppressor mutations (Truong and Boeke 2017; Haase *et al*. 2019). Individual adapted strains were isolated, and suppressor mutations were identified in a range of cellular processes, including subunits of outer kinetochore complexes. More recently, we showed that centromeric function is disrupted in histone-humanized yeast, resulting in declustered kinetochores and chromosomal instability (Haase *et al*. 2023). Furthermore, we characterized the DAM1/DASH suppressor mutants, in particular *dad1^E50D^*, and found that they prevent aneuploidy in histone-humanized strains by weakening kinetochore-microtubule attachments (Haase *et al*. 2023).

Given the centromere and kinetochore dysfunction in histone-humanized yeast, we explored whether addition of human CENP-A would alleviate the phenotype. We initially hypothesized that if addition of CENP-A (or a Cse4–CENP-A chimera) improved humanized centromeric nucleosome function, it would in turn expedite quality control mechanisms at the kinetochore in histone-humanized yeast. This is expected to improve humanization capabilities and potentially provide the basis for sequentially humanizing other kinetochore complexes, chromatin, or other cellular functions in future studies.

We show that CENP-A or yeast–human chimeras cannot replace Cse4, even in the context of human histones (hHs). Our data suggest that human CENP-A can be incorporated into budding yeast chromatin but does not support native kinetochore function. CENP-A overexpression studies in WT cells reveal aberrant kinetochore function and cell cycle progression. High-throughput genetic screening suggests that specific evolutionary-conserved chromatin remodelers are involved in the CENP-A overexpression phenotype. Furthermore, we show that chimera overexpression exhibits synthetic lethality with deletions of conserved kinetochore subunits and known centromere/kinetochore regulators.

Moreover, we explore the extent to which the outer kinetochore can be humanized by testing each subunit of the NDC80c (Ndc80, Nuf2, Spc24, and Spc25), individually or in various combinations, as well as all 4 simultaneously. Regrettably, we were unable to reproduce previously published finding showing replacement of yeast Ndc80 by its human counterpart (Zheng *et al*. 1999) nor any of the other subunits, despite multiple attempts using several humanization strategies. We discuss these incompatibilities in the context of previous yeast complementation studies. Together, these observations highlight the difference between the centromeres and kinetochores of humans and budding yeast but also underscore the underappreciated similarities in epigenetic regulation of centromeric function.

## Materials and methods

### Yeast methods and growth conditions

Yeast strains and plasmids used in this study are listed in Supplementary Tables 1 and 2, respectively.

Yeast strains were grown at 30°C in either yeast peptone dextrose medium (YPD; 1% yeast extract, 2% bacto-peptone, and 2% dextrose) or synthetic complete (SC) media containing 2% dextrose or 2% galactose/1% raffinose (Gal/Raf) unless otherwise stated and, depending on the plasmid being selected for, in dropout media lacking the relevant aa. Yeast CEN/ARS plasmids were used in all cases and edited using Gibson cloning in *Escherichia coli* or in vivo gap-repair cloning technique, which combines a linearized plasmid with PCR or synthesized DNA products utilizing homologs recombination in yeast. All yeast transformations were carried out using the lithium acetate method. All plasmids generated were verified by Sanger or nanopore sequencing. All PCR products were generated using Q5 polymerase (New England Biolabs, USA). All yeast strains and plasmids used in this study are available upon request.

### Yeast humanization assays

#### CEN nucleosome humanization

The histone shuffle *dad1^E50D^* strain (yMAH700), which lacks the core genomic histones and is maintained by single set of episomal yHs, was used as a positive control and to generate a new strain for Cse4 nucleosome swapping. To delete *CSE4*, we first adapted the yHs Superloser plasmid (pDT139, *URA3* marker) to contain the *CSE4* sequence. To minimize genomic integration and plasmid recombination, we designed the Superloser plasmid with a recoded *CSE4* (*rcCSE4*) sequence controlled by the orthologous promoter and terminator from the related species *Saccharomyces eubayanus* to create a yHs + *CSE4* Superloser plasmid (pGOL011). Next, we deleted *CSE4* from the genome by replacing it with the *HIS5MX6* cassette in yMAH700 containing pGOL011 (yGOL001) to create the CEN nucleosome shuffle strain (yGOL014). We also used a “WT” histone shuffle strain (yDT67) to make a CEN nucleosome shuffle strain without the *dad1^E50D^* mutation (yGOL187) using the same approach. We adapted the histone humanization plasmid (pDT109; *TRP1* marker), which contains the core hHs, to include the human CENP-A sequence codon-optimized for expression in yeast, and all the genes were controlled by corresponding native *S. cerevisiae* promoters and terminators, to create the hHs + CENP-A plasmid (pMAH464). The same approach was used to generate hHs + chimera plasmids. Then, the new CEN nucleosome shuffle strain was transformed to contain both the counter-selectable yHs + *CSE4* Superloser (pGAL1-CEN and 2x*URA3*) and the hHs + CENP-A or chimera (*TRP1*) plasmids. The Superloser plasmid can be destabilized by growing cells in media containing galactose, since the high-expression *GAL1* promoter is adjacent to the CEN sequence. Therefore, before plating various volumes of the cell cultures on the counter-selectable solid media containing 5-FOA, we grow them in SC–Trp Gal/Raf liquid medium for 24–48 h. Before plating onto SC–Trp 5-FOA, we measure the optical density (OD_600_) of the cultures (see Supplementary Table 3 for details of the culture ODs and volumes plated). We incubated the agar plates up to 2 months at 30°C in a sealed plastic box containing damp paper towels to prevent drying out the plates. We counted colonies after 20 days of growth and excluded large colonies that were visible within 3 days from further analysis to verify the loss of yeast histones by PCR genotyping as previously described (Haase *et al*. 2019, 2023). Humanized colonies per cells plated or “humanization frequency” was calculated by dividing the number of colonies counted by the total number of cells plated. To represent these values as “humanization rate per billion cells plated,” we multiplied the frequency number by 1 billion. Only plates that contained true positive humanized colonies verified by PCR to have lost yHs and *rcCSE4* were used to determine the humanization frequency. In case of the positive control histone humanization experiments with the *yHsΔ dad1^E50D^CSE4+* shuffle strain, only colonies that had retained hHs and CENP-A or chimeras and lost yHs and *rcCSE4* were determined to be humanized.

#### NDC80c humanization

To generate NDC80c shuffle strains, we deleted the coding sequences of *NDC80*, *NUF2*, *SPC24*, and *SPC25* from the genome of WT BY4741 using CRISPR/Cas9 genome editing as previously described (Haase *et al*. 2023). A targeting guide RNA plasmid was co-transformed with a donor template containing ∼40 bp upstream and downstream homology sequences flanking the relevant open reading frame into a strain expressing Cas9 (Cas9 plasmid, pNA0519, and sgRNA-expressing plasmids appropriate for each deletion). We verified successful editing by PCR genotyping for each individual deletion. To generate the complete deletion of the 4-subunit NDC80c, the same method was used sequentially until all subunits were deleted. We also made every combination of double deletions and a few triple deletions (see Supplementary Tables 1 and 2 for complete list of strains and plasmids, respectively). Since the genes encoding the NDC80c subunits are essential, the viability of the deletion strains was maintained by generating a Superloser plasmid containing the native NDC80c sequences (yNDC80c; pGOL087). To avoid plasmid recombination, we recoded the yeast gene sequences and used the related *S. eubayanus* promoters and terminators. The *S. eubayanus* promoters expressed the yNDC80c genes sufficiently as seen by the viability of the shuffle strains lacking the genomic yNDC80c subunits (*yNDC80cΔ*) containing the Superloser on synthetic media lacking uracil (Fig. 5). For the humanization assay, we engineered a human NDC80c (hNDC80c) plasmid based on pRS415 (*LEU2*) and cloned in yeast codon-optimized human NDC80c coding sequences controlled by *S. cerevisiae* promoters and terminators (pGOL081). We also generated plasmids containing individual subunits of hNDC80 (pGOL103-107). Next, we used 3 strategies to complement yeast NDC80c subunits with human orthologs (Supplementary Fig. 5a).

In the first strategy, we used the conventional Superloser double-plasmid shuffle assay as described above. In order to summarize and highlight our negative results, instead of only plating various volumes of the cell cultures on 5-FOA media, we also included serial dilution spot assays using the cultures and spotted onto solid SC media with and without 5-FOA using otherwise the same protocol as described earlier (Fig. 5; Supplementary Fig. 5). As before, we verified whether colonies had lost the yNDC80c genes using PCR genotyping. We note that we observed far fewer spontaneous 5-FOA-resistant colonies (which all turned out to retain the recoded yNDC80c genes) in the NDC80c humanization assays compared with the CEN nucleosome humanizations assays, which could suggest that centromere or chromatin disruption by the CEN nucleosome swapping creates a situation where plasmid recombination frequency is increased. However, further research is required to understand this curiosity.

In the second strategy, instead of deleting the yeast genes, we replaced them using CRISPR/Cas9 by utilizing the human ortholog sequences with *S. cerevisiae* promoters and terminators as donor templates and viability was maintained by the yNDC80c Superloser plasmid as in the first strategy. Thus, the hNDC80c *LEU2* plasmids were not required for this assay. However, the following steps were performed as in strategy 1, but since no plasmid selection was required, we instead used YP Gal/Raf liquid medium for the precultures and plated onto solid SC 5-FOA medium.

The third strategy was performed using CRISPR/Cas9 editing to replace individual NDC80c genes with the human orthologs as in strategy 2, but omitting the yNDC80c Superloser plasmid to maintain viability, and PCR genotyped the resulting transformants to verify successful complementation. However, for each of the 4 gene swaps, every transformant tested contained both the endogenous sequence and the human sequence, suggesting ectopic genomic localization, aneuploidy, or genome duplication. However, further studies are required to determine the possible genomic alterations in these transformants.

### Yeast growth spot assays

Overnight yeast cell cultures incubated at 30°C in the appropriate medium containing 2% Dex were diluted in fresh medium and incubated until mid-log phase (OD_600_ = 0.5–0.8). Cell density was measured and adjusted so that each culture had equal OD_600_. Serial dilutions (1:10) were performed and spotted onto appropriate solid media and incubated at 30°C for 3 days unless otherwise stated.

### High-throughput genetic interaction screens

The genome-wide genetic interactions screens were performed using the selective ploidy ablation (SPA) methodology (Reid *et al*. 2011). To this end, we generated conditional overexpression plasmids containing a *LEU2* selectable marker and DNA sequences of CENP-A (pGOL017), CENP-A^W86R^ (pGOL031) mutant, or chimera C (pGOL028) driven by the *GAL1* promoter. Using SPA, we transferred the plasmids, as well as an empty control plasmid (pAV115) into an array of the yeast deletion collection, which contains ∼4,800 nonessential knockout strains (Winzeler *et al*. 1999), as described previously (Fig. 4a; Ólafsson and Thorpe 2020; Reid *et al*. 2016). The assay was performed using a semiautomatic robotic pinning system, ROTOR HDA (Singer Instruments, UK), and rectangular agar plates containing the deletion collection previously arrayed as 384 different strains in quadruplicate per plate, i.e. at 1,536 colony density. Each incubation step was performed at 30°C. The final SC–Leu 2% Gal 5-FOA agar plates of the assay were incubated for 3–5 days and imaged using a ScanMaker 9800XL Plus (Microtek International) plate scanner.

### Screen data analysis

The colonies were analyzed using colony quantification software (Dittmar *et al*. 2010; Klemm *et al*. 2022). Typically, we would use a cutoff of *Z*-score of +/− 1 or 2 which are calculated from colony size ratios between control and experiment strains and typically normalized to the plate median value; however, in the case of CENP-A overexpression where almost everything on each plate was dead, this became an issue. We therefore used a more stringent cutoff using normalized growth ratio of >1.4, meaning that deletion strains overexpressing CENP-A that were 40% larger or more compared with the plate median colony size are considered positive interactions or suppressors, and we only considered interactions with *P*-values below 0.05. For the chimera C genetic screen, we used a more typical cutoff of *Z*-score >1.5 and this produced a list of negative interactions all of which have log growth ratios of 0.4 or higher, which is a conservative cutoff (Ólafsson and Thorpe 2018) and is easily visible by eye. Colonies that grew poorly with the empty control plasmid were excluded from the analysis. Gene ontology (GO) enrichment analysis was performed on these data sets using the webtool ShinyGO (v 0.77).

### Flow cytometry

We used a flow cytometry protocol as previously described (Haase *et al*. 2023). Before collecting and fixing cells for flow cytometry analysis, we induced overexpression for 6 h by shifting asynchronous early log-phase WT cell cultures grown in synthetic medium containing 2% raffinose to a medium containing 2% galactose and 1% raffinose. We collected log-phase cultures by centrifugation and resuspended in 1.5 mL of water. Next, we fixed and permeabilized the cells by slowly adding 3.5 mL of 100% ethanol and left overnight at −20°C. Then, we pelleted the cells and washed 3 times with water. To remove contaminating RNA, we centrifuged and resuspended the samples in an RNAse A solution (15 mM NaCL, 10 mM Tris pH 8.0, 0.1 mg/mL RNAse A) and incubated at 37°C overnight. Next, we pelleted the cells and resuspended in 50 mM Tris and mixed 0.5 mL of processed cells with 0.5 mL of SYTOX Green stain [2 µM SYTOX Green (Thermo Fisher cat. S7020) in 50 mM Tris pH 7.5] and incubated for 1 h at 4°C in the dark. Finally, we pelleted the cells and resuspended in 1 mL of Tris pH 7.5 and sonicated. Flow cytometry analysis was performed on the BD Accuri C6 flow cytometer, and the data were analyzed using FlowJo software (v10.0.7).

### Cell cycle and kinetochore analysis using fluorescence microscopy

Live-cell imaging was performed with exponentially growing yeast cultures in the appropriate SC media supplemented with 100 mg/mL of adenine to minimize autofluorescence. Prior to imaging, the cells were embedded in 0.7% low-melting point agarose dissolved in the growth medium used. The cells were imaged using the EVOS M7000 microscope (Olympus X-APO 100X Oil, 1.45NA/WD 0.13 mm oil objective). Images were processed with ImageJ and Icy image-processing software.

For the cell cycle analysis of cells containing fluorescently tagged kinetochore protein (i.e. Ndc80-GFP) and overexpression plasmid, overnight cultures growing in 2% Raf/0.1% Dex SC–Leu + Ade medium at 30°C were resuspended in fresh SC–Leu + Ade 2% Raf medium and incubated until mid-log phase (OD_600_ = 0.5–0.8) then resuspended in SC–Leu2 + Ade 2% Gal/1% Raf medium and incubated for 5 h before imaging. The cell cycle stages were estimated based on mother-bud cell morphology and kinetochore status, i.e. 1 or 2 foci, in proximity or separated. Unbudded cells with a single kinetochore focus were categorized as G1 cells. Cells with small buds and single kinetochore focus were considered as S-phase. Medium- or large-budded cells with 2 kinetochore foci in close proximity (<1.5 μm) were considered metaphase cells. Cells with 2 kinetochore foci, 1 in the mother and the other in the daughter, were classified as anaphase or telophase cells. The number of cells from multiple field-of-views counted from a single experiment is indicated as n. Fluorescence intensities of kinetochore foci were quantified using an ImageJ tool, FociQuant (Ledesma-Fernández and Thorpe 2015), and the Spot detector tool in Icy (v 2.1).

## Results

### Human CENP-A cannot replace yeast *CSE4* and is incompatible with histone humanization in *S. cerevisiae*

To swap out Cse4 for CENP-A, we used an improved version of the dual-plasmid shuffle assay “Superloser” (Fig. 1b and Materials and methods; Haase *et al*. 2019). We engineered a counter-selectable Superloser plasmid containing yHs as previously reported (Truong and Boeke 2017; Haase *et al*. 2019, 2023), further including *CSE4* (Supplementary Fig. 1a). We introduced this Superloser plasmid into a histone shuffle strain from which core histone loci and *CSE4* were deleted (*yHsΔ cse4Δ*). This new “CEN nucleosome” shuffle strain was transformed with a selectable humanization plasmid containing human core histones H3.1, H4, H2A, and H2B (hHs) and CENP-A. Plasmid shuffling works by selecting for a desired plasmid (humanization plasmid, *TRP1*) and against the plasmid to be lost (Superloser, 2× *URA3*), by growing cells in media lacking tryptophan (SC–Trp) and supplemented with 5-FOA, which is toxic to cells containing *URA3* (Boeke *et al*. 1987). We generated a CEN nucleosome shuffle strain, also including positive and negative controls (Fig. 1c), in a WT as well as in the *dad1^E50D^* background, which has improved histone humanization frequency (Truong and Boeke 2017; Haase *et al*. 2019, 2023). After 7–20 days’ growth, we isolated and identified successfully humanized clones (Supplementary Fig. 1b and c and Table 3).

No clones with successful replacement of Cse4 with CENP-A were identified, even after 2+ months of growth, whereas we successfully isolated histone-humanized colonies with the positive control (hHs plus *CSE4*) within 20 days (Fig. 1e; Supplementary Fig. 1b–d). Despite significant enhancement of the histone humanization rate conferred by the *dad1^E50D^* mutation, we were also unable to humanize the CEN nucleosome in the *dad1^E50D^cse4Δ* histone shuffle strain, in contrast to the positive control plasmid containing hHs and *CSE4* (Fig. 1c and e; Supplementary Fig. 1d). This was expected because Cse4 has an extended 135-aa N-terminal tail compared with the 44 aa of CENP-A, which in yeast is regulated by multiple posttranslational modifications (PTMs) (Hewawasam *et al*. 2010; Ranjitkar *et al*. 2010; Samel *et al*. 2012; Au *et al*. 2013; Boeckmann *et al*. 2013; Mishra *et al*. 2015, 2019; Ohkuni *et al*. 2016; Hoffmann *et al*. 2018; Mishra and Basrai 2019), and contains the “essential N-terminal domain.”

We hypothesized that the yeast Cse4 N-terminal tail is needed for assembly of native kinetochore components in humanized yeast, especially because these PTM sites are crucial for CEN nucleosome function, and END is important for interaction with the COMA subcomplex of the CCAN, providing a direct link between the centromeric nucleosome and the outer kinetochore KMN network (Fischböck-Halwachs *et al*. 2019). To this end, we generated various Cse4–CENP-A chimeras containing different extents of Cse4 N-terminal tail (Cse4^N-tail^) genetically linked to CENP-A at the N-terminus, with and without the CENP-A^N-tail^ (Fig. 1d), 2 chimeras containing END plus upstream residues (61 aa in total; Cse4^N61^) fused to either CENP-A with a truncated N-terminus (chimera A) or to full-length CENP-A (chimera B), and chimera C, containing the whole Cse4^N-tail^ fused to the histone-fold domain (HFD) of CENP-A (Cse4^N^-CENP-A^HFD^; Fig. 1d). However, neither the END nor the entire Cse4^N-tail^ fused to CENP-A was sufficient to replace Cse4 (Fig. 1e).

In our assays, we occasionally observed colonies forming in 2–3 days at a low frequency. In every case of genotyping, these clones were found to retain a Superloser plasmid that lost *URA3* or the yHs genes were recombined into the humanization plasmid. Infrequently, we also observed colonies forming after 7+ days of growth (Supplementary Fig. 1f). Genotyping these colonies in both *dad1^E50D^ CSE4+* and *dad1^E50D^cse4Δ* histone shuffle strains showed that they had humanized the core histones and lost CENP-A or the chimera but retained episomal *CSE4* (Supplementary Fig. 1e and f); restriction digestion analysis and nanopore sequencing indicated plasmid recombination. We never isolated *cse4Δ* clones (WT or *dad1^E50D^*) with yeast histones and CENP-A or chimera.

In *S. cerevisiae*, “Loop 1” (L1) of Cse4^HFD^ is important for CBF3c interaction (Guan *et al*. 2021), in particular 3 residues, K172, D173, and Q174, which are not conserved in CENP-A L1 (highlighted in Fig. 1e insert and aa sequence alignment of HFD is shown in Supplementary Fig. 1g). Also important for this interaction is the R120 residue of yeast H2B (K117 in human H2B; highlighted in Fig. 1e insert; Guan *et al*. 2021). Thus, we asked whether swapping back these residues would be sufficient for humanization but found that they were not (chimera D and hH2B^K117R^ in Fig. 1d–e). To address whether potentially low protein levels of the chimeras were preventing humanization or whether simultaneous histone/CenH3 humanization was too disruptive for cell growth, we also performed a direct Cse4 complementation shuffle assay in *cse4Δ* cells containing highly expressed pGAL1-chimeras, without deleting native histones. However, this was not the case, as *cse4Δ* strains expressing chimeras did not survive, in contrast to pGAL1-*CSE4* expression (Supplementary Fig. 1h).

Next, we dissected the HFD and generated additional swap-back chimeras containing various sections of Cse4^HFD^ swapped back into CENP-A^HFD^ of chimera C. However, repeating the humanization assay with these additional chimeras (chimeras E–J), we did not identify any humanized colonies in either *cse4Δ* shuffle strains and did not improve histone humanization in the *dad1^E50D^CSE4*+ shuffle strain (Fig. 1e; Supplementary Fig. 1d and f). Taken together, our data extend the conclusions of Stoler *et al*. (1995) showing that both native human CENP-A and Cse4–CENP-A chimeras are incompatible with the budding yeast centromere even in the context of human core histones and suggest there are fundamental functional differences between the centromeric nucleosomes of the 2 species.

### Overexpression of CENP-A is lethal in WT cells, and CENP-A protein escapes Cse4 regulatory machinery

**Fig. 2.**
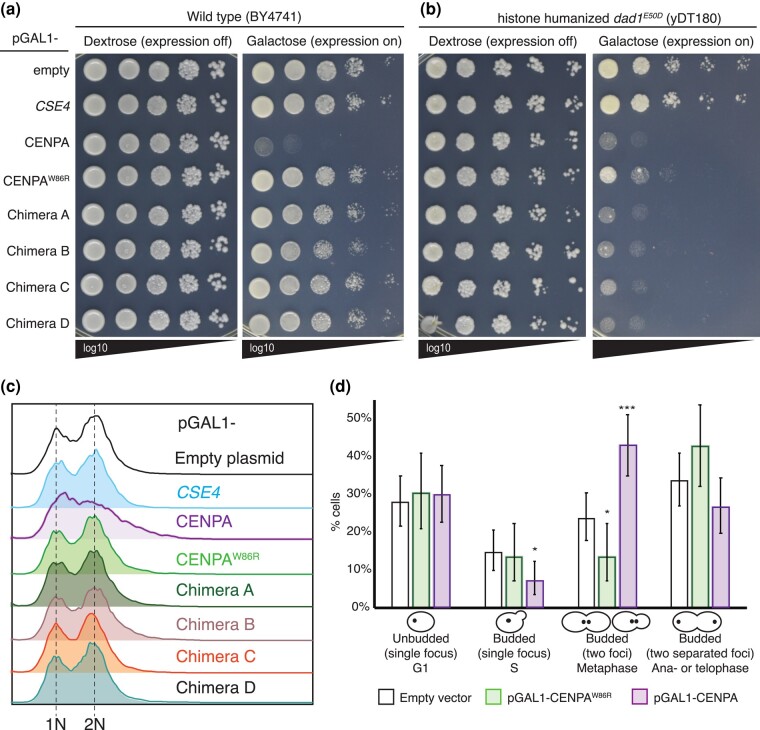
Overexpression of human CENP-A is lethal in WT yeast and perturbs mitotic progression. a) A spot assay showing that CENP-A overexpression is lethal in WT cells in contrast to controls. Log-phase cultures of WT strain (BY4741) with the indicated plasmids were diluted to the same OD_600_ level, and 10-fold serial dilutions were prepared and spotted onto media containing dextrose (expression off) and galactose (expression on). b) Same as panel a), but the indicated plasmids were transformed into a stable histone-humanized dad1^E50D^ strain (yDT180). c) Cell cycle analysis by flow cytometry of WT cells containing the indicated plasmids. Asynchronous log-phase cultures were grown in media containing galactose for 6 h before cell fixing and DNA staining with SYTOX Green and DNA content quantification (see Materials and methods for details). The experiment was performed in duplicate with identical results. d) Cell cycle analysis by fluorescence microscopy of WT cells containing an empty vector (*n* = 190), pGAL1-CENP-A^W86R^ mutant (*n* = 89), and pGAL1-CENP-A (*n* = 154) plasmids and GFP-tagged Ndc80, an outer kinetochore subunit of the KMN network. Asynchronous log-phase cultures were grown in media containing galactose for 5 h before imaging. Mother-bud cell morphology and kinetochore foci status were used as proxy for cell cycle phases. For example, large-budded cells with 2 kinetochore foci in close proximity were categorized as metaphase cells (see Materials and methods for details). Fisher’s exact statistical test; *P*-values *P < 0.05; ***P < 0.0005. Error bars indicate 95% binomial confidence intervals (CI).

We explored the CENP-A phenotype in more detail and assessed the effect of CENP-A overexpression on cell growth using a serial dilution spot assay (Supplementary Fig. 1i). The conserved tryptophan residue CENP-A^W86^ (Cse4^W178^) in L1 is reportedly crucial for centromere targeting in human cells (Shelby *et al*. 1997) and participates in a hydrophobic patch that promotes histone H4 interaction (Sekulic *et al*. 2010). Mutation of this residue to a hydrophilic or charged residue prevents CENP-A nucleosome incorporation (Shelby *et al*. 1997) and for Cse4 can cause lethality or temperature sensitivity in *S. cerevisiae* (Keith *et al*. 1999). Thus, as a control, we generated a mutant pGAL1-CENP-A^W86R^ as well as an empty vector control. Together with pGAL1-CENP-A, we introduced these plasmids into the histone shuffle strain and found that CENP-A overexpression prevented growth (Supplementary Fig. 1i). We considered the possibility that the lethality was specific to the *dad1^E50D^* histone shuffle strain. To rule this out, we tested this in a WT BY4741 strain and used chimera and *CSE4* overexpression plasmids, which are all viable in WT cells, as controls. Strikingly, we found that CENP-A overexpression was also lethal in WT cells, in contrast to the overexpression of *CSE4*, Cse4–CENP-A chimeras, and CENP-A^W86R^ mutant (Fig. 2a). Since overexpression of the mutant CENP-A^W86R^ was viable, this suggests that WT CENP-A can form nucleosomes with yeast histones. However, we suspected that the lack of compatibility between human CENP-A and yeast centromeric nucleosomes was the main driver of the observed phenotype. Therefore, we assessed this in a stable histone-humanized *dad1^E50D^* yeast strain (Truong and Boeke 2017). We found that CENP-A and, to our surprise, also the chimeras, were lethal in this case (Fig. 2b). This suggests that CENP-A-dependent lethality is not a consequence of CENP-A's failure to incorporate into yeast chromatin but rather results from its successful incorporation into both native and humanized nucleosomes. Additionally, this suggests that kinetochore dysfunction in histone-humanized yeast may be further compromised by chimera overexpression. Interestingly, overexpression of the CENP-A^W86R^ mutant was toxic to the humanized histone strain (Fig. 2b).

Cells control excessive CenH3 levels by ubiquitylation, marking it for proteasomal destruction (Collins *et al*. 2004; Hewawasam *et al*. 2010; Ranjitkar *et al*. 2010; Cheng *et al*. 2017; Dong *et al*. 2021). The E3 ligase Psh1 is a well-characterized Cse4 regulator, and its deletion sensitizes yeast to *CSE4* overexpression (Hewawasam *et al*. 2010; Ranjitkar *et al*. 2010). Cse4 contains 4 lysine residues reportedly ubiquitylated by Psh1 (K131, K155, K163, and K172; Hewawasam *et al*. 2010), of which only K155 is conserved in CENP-A (Supplementary Fig. 1g). Therefore, we speculated that Psh1 failed to mark CENP-A for proteolysis, resulting in a phenotype similar to *CSE4* overexpression in *psh1Δ* cells. We tested this hypothesis by repeating the overexpression spot assay using the *psh1Δ* strain. Since CENP-A overexpression is completely lethal on media containing 2% galactose/1% raffinose, we also performed the assay on 0.1% glucose/2% raffinose. This revealed that the deletion of *PSH1* did not further sensitize cells to CENP-A overexpression (Supplementary Fig. 2a). Since chimera overexpression did not perturb WT cellular growth and the addition of 61-aa Cse4^N-tail^ fragment to full-length CENP-A rescued CENP-A lethality (chimera B in Fig. 2a), we wondered whether this was sufficient to make CENP-A detectible by Psh1. Therefore, we also tested chimera overexpression in the *psh1Δ* strain and found that it did not affect growth (Supplementary Fig. 2b). This suggests that Psh1 is unable to regulate CENP-A and the chimeras.

Many important posttranslational modifiers of Cse4 have been identified, including multiple kinases and ubiquitin ligases, all of which target the N-terminal tail of Cse4 (Au *et al*. 2013; Boeckmann *et al*. 2013; Ohkuni *et al*. 2016, 2018; Mishra *et al*. 2019, 2021; Mishra and Basrai 2019). This may explain the observed rescue of CENP-A overexpression–dependent lethality by the addition of the Cse4^N-tail^. It has been reported that 2 additional lysines (K215 and K216) in the C-terminus of Cse4 are regulated by sumoylation and ubiquitylation in a process important for Cse4 deposition into chromatin and overexpression of a *cse4^K215,216^* mutant was not toxic in *psh1Δ* cells (Ohkuni *et al*. 2020). Notably, CENP-A differs significantly from Cse4 at this motif (see highlighted in Supplementary Fig. 1g), and unlike Cse4, it lacks sumoylation consensus sites (Chang *et al*. 2018). We already performed humanization assay with a chimera that has the 4 lysines targeted by Psh1 (chimera H), but the accompanying HFD sections were swapped back in this chimera; hence, this was perhaps too extensive an alteration for compatibility with otherwise humanized nucleosomes. Thus, we tested another chimera with just the Psh1-targeting lysine residues swapped back in the CENP-A HFD and the Cse4 C-terminal SUMO motif swapped back (chimera K) and found that it was insufficient to replace Cse4 and histone humanization frequency was similar to the other chimeras in the *yHsΔ dad1^E50D^CSE4+* shuffle strain (Fig. 1e; Supplementary Fig. 1d).

Both Cse4 and CENP-A contain an important “CENP-A targeting domain” (CATD), which shares only ∼50% identity and spans about 40 aa in the HFD and includes L1 (Supplementary Fig. 1g). CATD is required for CenH3 incorporation into centromeres (Vermaak *et al*. 2002; Black *et al*. 2004, 2007). Moreover, Psh1 requires CATD to target Cse4 (Ranjitkar *et al*. 2010; Zhou *et al*. 2021). Therefore, we attempted to humanize the CEN nucleosome, using a chimera with the Cse4 CATD swapped back (chimera L in Fig. 1d). However, this chimera too was unable to humanize the CEN nucleosome (Fig. 1e; Supplementary Fig. 1d and f). These findings indicate that Cse4 cannot be excessively altered for humanization and that the overall aa sequence is required for appropriate regulation, structure, and centromere function, even in the context of human core histones.

### CENP-A overexpression disrupts cell cycle progression and the kinetochore in WT cells

Mislocalized CenH3 can form ectopic kinetochores at noncentromeric euchromatin in metazoans and is likely a driver of aneuploidy and chromosome segregation abnormalities that result in chromosomal instability (Heun *et al*. 2006; Shrestha *et al*. 2017). However, whether ectopic kinetochore protein recruitment forms functional kinetochores remains debatable (Van Hooser *et al*. 2001; Bodor *et al*. 2014; Shrestha *et al*. 2017; Dong *et al*. 2021). Excessive protein levels of Cse4 causes its misincorporation into regions associated with high histone turnover, such as high-expression promoters and rDNA (Camahort *et al*. 2009; Hildebrand and Biggins 2016; Hewawasam *et al*. 2018). This further results in abnormal expression of a variety of genes, which likely affects general cell homeostasis and cell cycle progression (Hildebrand and Biggins 2016; Hewawasam *et al*. 2018).

We further characterized the CENP-A overexpression phenotype by assessing cell cycle progression by measuring DNA content using flow cytometry. After inducing overexpression for 6 h, we observed that controls and chimeras did not affect DNA content, whereas CENP-A-overexpressing cells showed abnormal G1 to G2/M transition, as seen by the lack of clear 1N and 2N peaks, and we noticed a slight increase in >2N DNA, as seen by the longer right spread-out pattern of the 2N peak, suggesting chromosomal instability (Fig. 2c).

**Fig. 3.**
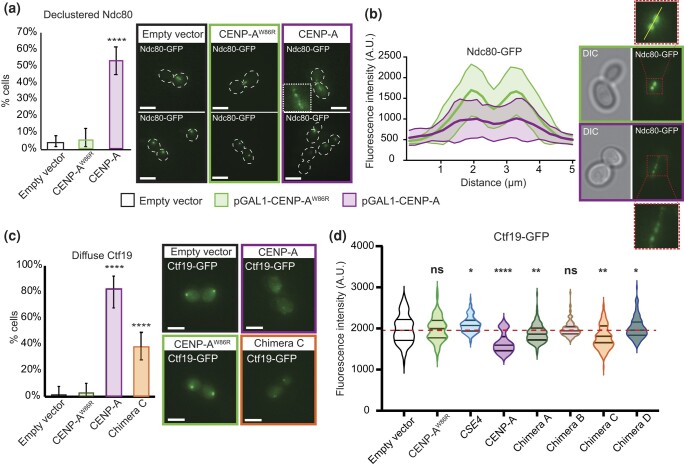
CENP-A overexpression disrupts the inner and outer kinetochore in yeast. a) The kinetochore foci of the Ndc80-GFP cells in Fig. 2d were analyzed using fluorescence microscopy, and abnormal declustered Ndc80-GFP foci were quantified. Fisher’s exact statistical test; *P*-values ****P < 10^−5^. Error bars indicate 95% binomial CI. Representative micrographs are shown on the right. Scale bars indicate 5 µm. b) The Ndc80-GFP signal in panel a) was also quantified by measuring the GFP intensity along the mitotic spindle. A 5-μm line with 30 points (illustrated by a yellow line in the inset on the top right) was used to include background signal and to cover the spread GFP signal in cells overexpressing CENP-A (40 randomly selected mitotic spindles were measured for each condition). The shadowed area indicates standard deviation. CENP-A-overexpressing cells have a more dispersed arrangement of Ndc80-GFP, as indicated by the flatter profile. Representative micrographs are shown on the right. Bottom right insert highlights the declustered Ndc80-GFP phenotype. c) Kinetochore foci analysis of WT cells containing the CCAN subunit Ctf19-GFP and the empty vector (*n* = 71), pGAL1-CENP-A^W86R^ (*n* = 67), pGAL1-Chimera C (*n* = 89), and pGAL1-CENP-A (*n* = 45) plasmids. Asynchronous log-phase cell cultures were grown in media containing 2% galactose for 5 h before imaging. Cells containing no or diffused Ctf19-GFP signal were quantified. Fisher’s exact statistical test; *P*-values ****P < 10^−5^. Error bars indicate 95% binomial CI. Representative micrographs are shown on the right. Scale bars indicate 5 µm. d) Violin plot showing fluorescence intensities of Ctf19-GFP kinetochore foci in cells containing empty vector (*n* = 107), pGAL1-CENP-A^W86R^ (*n* = 112), pGAL1-CSE4 (*n* = 110), pGAL1-CENP-A (*n* = 40), pGAL1-chimera A (*n* = 129), pGAL1-chimera B (*n* = 113), pGAL1-chimera C (*n* = 144), and pGAL1-chimera D (*n* = 76) plasmids. Culture conditions are the same as those in panel c). Statistical significance was evaluated using unpaired 2-tailed Student's *t*-test; *P*-value *P < 0.05; **P < 0.005; ****P < 10^−5^. Cells containing no detectable or very diffused Ctf19-GFP signals were excluded from the analysis.

Next, we analyzed the cell cycle using live-cell fluorescence microscopy by utilizing the overexpression plasmids in WT cells containing Ndc80, an outer kinetochore subunit of the KMN network tagged with green fluorescent protein (GFP). After 6 h of induction, cells overexpressing CENP-A accumulated in metaphase compared with controls, suggesting mitotic defects (Fig. 2d). Many CENP-A-overexpressing cells had abnormal Ndc80-GFP foci, prompting us to investigate whether CENP-A overexpression disrupted kinetochore function. To assess effects on the kinetochore, we analyzed kinetochore foci using fluorescence microscopy in cells containing different GFP-tagged kinetochore proteins, representing inner and outer kinetochores. Strikingly, ∼50% of the cells containing Ndc80-GFP displayed a declustered Ndc80 phenotype when CENP-A was overexpressed, whereas control cells containing an empty plasmid or overexpressing CENP-A^W86R^ did not (Fig. 3a and b; Supplementary Table 4). Similarly, CENP-A-overexpressing cells containing GFP-tagged Mtw1, a subunit of the MIND^MIS12^ subcomplex, showed a declustered kinetochore phenotype in ∼40% of cells, and chimera C overexpression showed an intermediate phenotype, or in ∼10% of cells (Supplementary Fig. 3a).

We also investigated the inner kinetochore and examined Ctf19-GFP, a nonessential subunit of CCAN, and Cep3-GFP, an essential subunit of CBF3c. In both cases, we observed a diffuse GFP signal in majority of cells overexpressing CENP-A (∼80% and ∼65%, respectively), instead of the 1–2 foci normally seen, in contrast to the empty vector and CENP-A^W86R^ controls (Fig. 3c; Supplementary Fig. 3b). Chimera C overexpression also displayed diffuse Ctf19-GFP and Cep3-GFP signals, but to a much lesser degree (∼40% and ∼30%, respectively). We further analyzed the Ctf19-GFP signal, including additional controls and chimeras (Fig. 3d). By quantifying fluorescence intensity of Ctf19-GFP foci, we discovered that CENP-A-overexpressing cells had significantly reduced Ctf19 foci intensity compared with empty vector control, interestingly, also chimeras A and C, although to a lesser extent (cells with a very diffuse signal and no foci were excluded from the analysis; Fig. 3d). Additionally, in contrast to Ctf19, we noticed that Cep3-GFP cells overexpressing CENP-A that contained foci (∼35%) had increased Cep3-GFP foci intensity (Supplementary Fig. 3c). These differences may be partially explained by the fact that Cep3, part of the CBF3c, is recruited in a Cse4-independent manner and binds directly to CDEIII centromeric DNA, whereas Ctf19 (part of CCAN) requires assembled centromeric nucleosomes and CCAN for recruitment. We also noted that the Cep3-GFP phenotype appeared qualitatively different from Ctf19-GFP, often appearing as large clusters/foci or bright nuclear signal, compared with 1–2 small foci in control cells, which can affect the intensity quantification in Supplementary Fig. 3c (see Supplementary Fig. 3b arrowheads and Discussion). Together, these observations indicate that CENP-A (and to a lesser degree chimera C) directly interferes with kinetochore function, consequently disrupting mitosis and cell cycle progression.

### Conserved SWI/SNF chromatin remodelers facilitate CENP-A incorporation into yeast chromatin

Several chaperones and chromatin modifiers have been implicated in centromere/kinetochore function (Baetz *et al*. 2004; Krogan *et al*. 2004; Vernarecci *et al*. 2008; Canzonetta *et al*. 2016). For example, the histone–chaperone CAF-1 and the INO80 chromatin remodeling complexes are reported to promote ectopic localization of Cse4 into euchromatin (Hildebrand and Biggins 2016; Hewawasam *et al*. 2018). Both reports showed that growth defects of *CSE4*-overexpressing *psh1Δ* cells were suppressed by deletions of the CAF-1 and INO80 subunits, *CAC2* and *NHP10*, respectively. We reasoned that the CENP-A phenotype we observed is partly due to the failure of yeast Cse4 regulatory mechanisms to safeguard against high CENP-A protein levels, thus enabling it to localize to noncentromeric regions. To examine this, we overexpressed CENP-A in *nhp10Δ* and *cac2Δ* cells to determine whether this would suppress this phenotype. Indeed, when we examined the growth phenotypes on 0.1% galactose/2% raffinose media, we observed suppression of growth inhibition; however, we did not notice a clear suppression on 2% galactose (Supplementary Fig. 2c), consistent with the results of previous studies (Hildebrand and Biggins 2016; Eisenstatt *et al*. 2021).

**Fig. 4.**
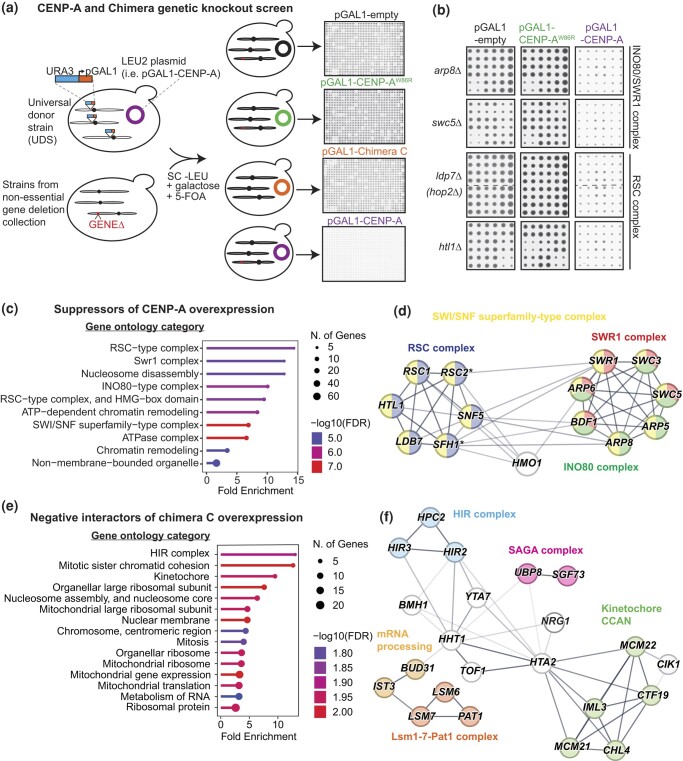
Identification of genetic interactions of CENP-A and chimera overexpression using genome-wide screening of nonessential gene deletions. a) Overview of the genetic screens. We used SPA to introduce the indicated plasmids into the whole nonessential gene knockout collection (see main text and Materials and methods for details). b) Example cropped images of arrayed colonies showing suppression of the pGAL1-CENP-A growth phenotype by deletions of INO80/SWR1 and RSC complex subunits. The images were captured after 5 days of growth at 30°C on synthetic media containing 5-FOA. c) GO enrichment analysis of CENP-A overexpression suppressors. d) Protein–protein interaction (PPI) network of CENP-A overexpression suppressors. The network was generated using the STRING database (Szklarczyk *et al*. 2023) and consists of a subset of the deleted genes identified as suppressors (see Supplementary Table 5 for the completed list). * indicates that these genes were not directly detected, but these 2 dubious open reading frames, *YLR358C* and *YLR322W/VPS65*, partially overlap with the essential RSC subunits *RSC2* and *SFH1*, respectively. Edge intensity that connects nodes indicates the confidence of the PPI (based on the STRING database): dark gray, high; light gray, low. Node color indicates different protein complexes. The size of the nodes or length of the edges do not reflect any quantifiable scores. e) GO enrichment analysis of negative genetic interactors of chimera C overexpression. f) PPI network of the negative interactors of chimera C overexpression. The PPI network shows a subset of negative interactors (see Supplementary Table 5 for the completed list). Edge intensity that connects nodes indicates the confidence of the PPI: dark gray, high; light gray, low. Node color indicates different protein complexes. The size of the nodes or length of the edges do not reflect any quantifiable scores.

This prompted us to investigate whether other factors were required for the observed phenotype. To examine this in an unbiased systematic manner, we screened for suppressors of CENP-A overexpression in a genome-wide collection of nonessential deletion strains (Fig. 4a; Winzeler *et al*. 1999; Reid *et al*. 2011, 2016; Ólafsson and Thorpe 2020). As expected, most of the colonies of the arrayed deletion strains overexpressing CENP-A did not grow, except for a few that visually stood out from the rest, in contrast to the control plates, where most strains grew (Fig. 4b). We carried out GO analysis of suppressors that formed colonies that were 40% or larger than the plate median colony size (114 gene deletions) of CENP-A overexpression (Fig. 4c; Supplementary Table 5). This set of genes was enriched for GO terms involved in biological processes, such as “chromatin disassembly” and “chromatin remodeling,” and the most highly enriched GO terms for cellular components included the SWI/SNF superfamily-type chromatin remodeling complexes, RSC, INO80, and SWR1 (Fig. 4c). It should be noted that this suppression was subtle (Fig. 4b) but verifiable (Supplementary Fig. 4a), perhaps a result of requiring 2% galactose in the SPA screen (instead of 0.1% galactose/2% raffinose, as observed in the spot assay suppression in Supplementary Fig. 2c), implying that our list of suppressors was undersaturated. Indeed, the suppressor screen did not identify the INO80c subunit *nhp10Δ*, nor CAF-1c subunits, but did identify deletions of the following INO80c and SWR1c subunits: *BDF1*, *ARP8*, *ARP5*, *ARP6*, *SWR1*, *SWC3*, and *SWC5* (Fig. 4b and d; Supplementary Fig. 4a and Table 5). It should be noted that the INO80 and SWR1 complexes share a number of subunits (Mizuguchi *et al*. 2004). Furthermore, we identified deletions of 4 RSC subunits, *RSC1*, *LDB7*, *HTL1*, and *SNF5* (Fig. 4b and d, Supplementary Fig. 4b and Table 5). These data generally conform to a previously published genome-wide suppressor screen of *CSE4* overexpression in *psh1Δ* cells (Eisenstatt *et al*. 2021; see Discussion), highlighting a conserved mechanism of CenH3 regulation and indicating that the CENP-A phenotype partly depends on chromatin remodeling factors important for centromere function.

### Genome-wide screening identifies synthetic dosage lethality between Cse4–CENP-A chimera overexpression and conserved Cse4 regulators

We included chimera C overexpression in the genome-wide screen to search for synthetic dosage lethal (SDL) interactions to further understand the overexpression phenotype (Fig. 4a). We found that SDL interactors of chimera C overexpression (155 gene deletions) were enriched for a variety of biological processes, such as mitosis, nucleosome assembly, and RNA metabolism (Fig. 4e). We also observed enrichment of cellular components, such as the ribosome, nuclear membrane, and kinetochore, including deletions of 5 CCAN subunits (*MCM22*^CENP-K^, *CTF19*^CENP-P^, *IML3*^CENP-L^, *MCM21*^CENP-O^, and *CHL4*^CENP-N^) of the inner kinetochore and deletions of 3 subunits of the centromere-associated HIR^HIRA^ histone–chaperone complex (*HIR1*, *HIR2*, and *HPC2*; Fig. 4e and f; Supplementary Table 5). Moreover, among the negative interactors of chimera overexpression with known centromeric functions were knockouts of the conserved AAA + ATPase *YTA7*^ATAD2/ANCCA^, histone subunits *HHT1*^H3^ and *HTA2*^H2A^, 3 members of the Lsm1-7-Pat1 mRNA-processing complex (*LSM6*, *LSM7*, and *PAT1*), and 2 subunits of the SAGA complex *UBP8* and *SGF73* (Fig. 4f). Notably, we did not identify SDL between chimera overexpression and *psh1Δ*, in agreement with our spot assay (Supplementary Fig. 2b). Nevertheless, these CEN-associated SDL interactors generally overlap well with the published SDL interactors of *CSE4* overexpression (Ciftci-Yilmaz *et al*. 2018).

We confirmed the SDL phenotype of chimera C in strains lacking CCAN and HIR complex subunits as well as in the *yta7Δ* strain using spot assays (Supplementary Fig. 4c and d). Moreover, we examined the overexpression of other chimeras in *the hir1Δ*, *hpc2Δ*, *and yta7Δ* strains and found that chimeras A, B, and C all produced SDL phenotypes that were much stronger than *CSE4* overexpression (Supplementary Fig. 4d). This suggests that deletion of these factors may exacerbate dysfunction of already compromised kinetochores in chimera-overexpressing cells. However, we found that deletion of *CBF1*, which is associated with reduced kinetochore function, did not produce SDL with chimera C overexpression (Supplementary Fig. 4c), and deletion of SAC components, which are sensitive to kinetochore dysfunction, was not found in our list of negative interactions (Supplementary Table 5). Overall, these data confirm the findings from published *CSE4* overexpression genetic interaction screens and highlight the important roles of Yta7, HIR, Lsm1-7-Pat1, and SAGA complexes in regulating CEN nucleosome function.

### Human outer kinetochore NDC80c subunits fail to complement

Our findings indicate that human CENP-A (and Cse4–CENP-A chimeras) conflict with yeast centromere function, disrupt the kinetochore, and likely generally disrupt chromatin. Evidently, this is an inadequate strategy for further humanization of chromatin-associated components, such as the kinetochore, in future experiments. Therefore, we sought to explore the similarity between the human and yeast outer kinetochore components using the same humanization approach, starting with the conserved NDC80c, a subcomplex of the KMN network.

Since it was reported that the human NDC80/HEC1 gene can fully complement yeast *NDC80* (Zheng *et al*. 1999), we investigated whether the 4-subunit NDC80c (Ndc80, Nuf2, Spc24, and Spc25) could be complemented by its human orthologs. We generated dual-plasmid shuffle strains by deleting NDC80c subunits from the genome using CRISPR/Cas9 (Supplementary Fig. 5a, strategy 1). Because these genes are essential, the strains were first transformed with a Superloser plasmid containing the 4 *S. cerevisiae* NDC80c gene sequences (yNDC80c) and then with the humanization plasmid containing human NDC80 subunits (hNDC80), all controlled by corresponding native yeast promoters.

**Fig. 5.**
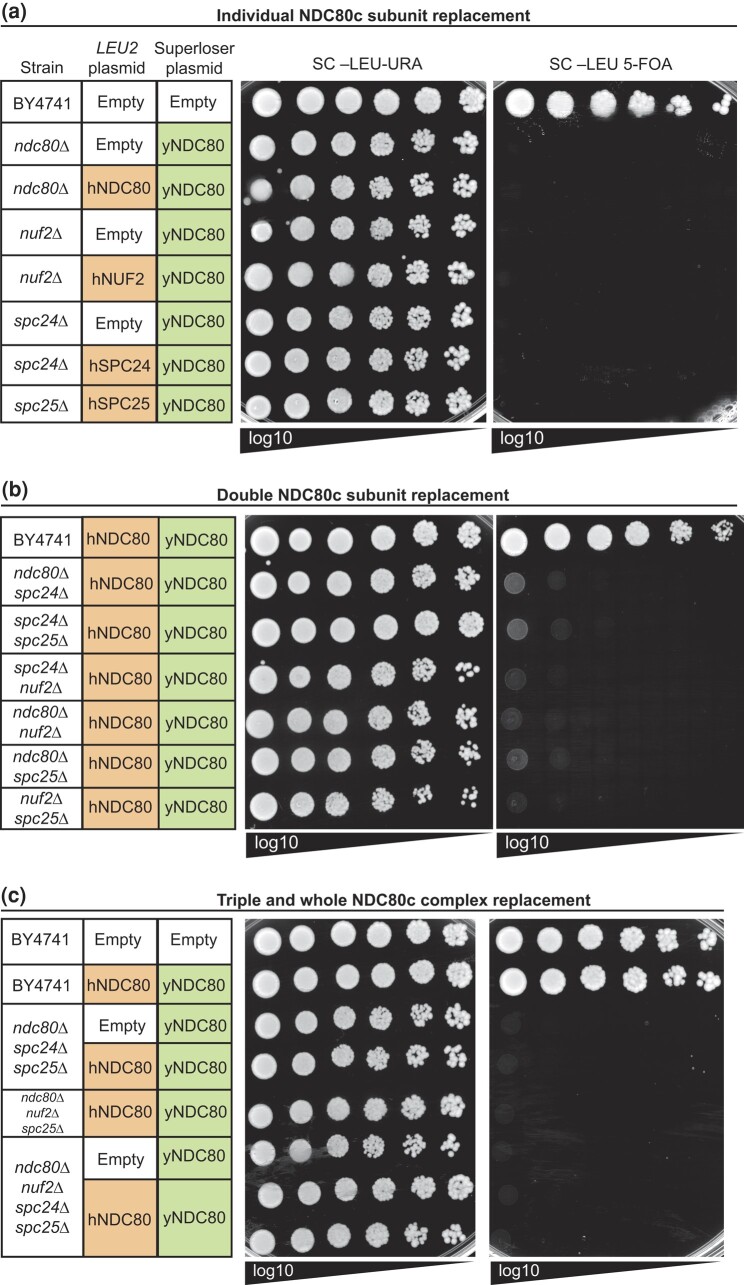
Complementation of the yeast NDC80c subunits by the human counterparts. a) Example complementation spot assay of individual replacements of yeast NDC80c subunits by episomal human orthologs. Complementation experiments were also performed by plating various amounts of cells (see Supplementary Table 6). b) Example complementation spot assay of double replacements of yeast NDC80c subunits by episomal human orthologs. c) Example complementation spot assay of triple and complete replacements of yeast NDC80c subunits by episomal human orthologs.

We performed dual-plasmid shuffle assays, as before, to replace each subunit individually (Fig. 5a), every binary combination of double replacements (Fig. 5b), 2 triple replacements, and all 4 subunits simultaneously (Fig. 5c). Surprisingly, we did not successfully identify any humanized colonies, despite multiple variations of plating on media containing 5-FOA (Fig. 5; Supplementary Table 6). In some cases, colonies arose but were eliminated by PCR screening as yeast recombinants (Supplementary Fig. 5e–g).

We separately attempted a single-plasmid shuffle approach by engineering new strains using CRISPR/Cas9 to integrate human subunits into the genomic loci in place of their yeast counterparts and sustained episomally by the Superloser plasmid containing yNDC80c (hNdc80c replacement strain; Supplementary Fig. 5a, strategy 2) to replace NDC80c components individually and in combination without evidence of complementation (Supplementary Fig. 5b–d and g and Table 6).

Finally, to rule out that plasmid shuffle methodology was generally inappropriate for replacing NDC80c subunits, we also used CRISPR/Cas9 to directly replace yeast *NDC80*, *NUF2*, *SPC24*, and *SPC25* by utilizing the DNA sequences of hNDC80c codon-optimized and flanked by *S. cerevisiae* promoters and terminators as donor templates (Supplementary Fig. 5a, strategy 3), but they could not be replaced this way either. Thus, the NDC80c or its subunits, including *NDC80*, could not be humanized notwithstanding the report by Zheng *et al*. (1999).

## Discussion

In this study, we tested the hypothesis that introducing human CENP-A or Cse4–CENP-A chimeras along with human canonical histones would improve histone humanization efficiency and facilitate kinetochore function in yeast with hHs. Our data suggest that human CENP-A is incompatible with kinetochore function in budding yeast. We discovered that CENP-A and chimeras were unable to replace Cse4: neither directly nor alongside humanization of the core histones. We also found that CENP-A expression reduced histone humanization frequency in the *dad1^E50D^CSE4+* histone shuffle strain (Fig. 1e; Supplementary Fig. 1d) and that overexpression was toxic and perturbed cell cycle and kinetochore function.

Cse4 complementation by orthologs from other fungal species has been successful, although phylogenetic distance of complementation is limited (Stoyan and Carbon 2004; Baker and Rogers 2006). Baker and Rogers used chimeras containing the Cse4 N-terminal tail from *S. cerevisiae* and HFD from the divergent fungus *Pichia angusta*. They found that chimeras that contained mostly *S. cerevisiae* Cse4^HFD^ complemented Cse4 and concluded that complementation success correlated with the extent of aa identity rather than with the presence or absence of any given element of the HFD secondary structure (Baker and Rogers 2006). This conclusion is corroborated by our data, and we note that *S. cerevisiae* Cse4^HFD^ shares more identity with *P. angusta* CenH3^HFD^ (∼69% identity) than with human CENP-A^HFD^ (∼58% identity). This is contrasted by a striking result indicating that CenH3 from *Plasmodium falciparum* (the parasite that causes malaria in humans) could functionally complement budding yeast temperature-sensitive Cse4 mutant strain at the nonpermissive temperature (Verma and Surolia 2013), despite lacking the Cse4^N-tail^ and the HFD sharing only ∼59% identity and less similarity (∼71%) than those of human (∼76%) and *P. angusta* (∼84%) (see Supplementary Fig. 1g for multiple-sequence alignment of the HFD).

It is possible that other hHs suppressor mutations, not tested here, would favor the replacement of Cse4 with CENP-A or full centromeric nucleosome humanization. Notably, many complementation studies, such as those that successfully complemented Cse4 mentioned above, were performed using temperature-sensitive mutant yeast genes complemented by episomal orthologs at nonpermissive temperatures. In these cases, it is possible that yeast mutant proteins have residual activity/function at these temperatures, which may be synergistically enhanced by the presence of nonnative orthologs. As noted by Hamza *et al.*, this can represent partial or indirect complementation and can be misleading (Hamza *et al*. 2015). Thus, for full complementation, deletion of the entire endogenous gene in question is appropriate.

### Possible mechanisms underlying CENP-A overexpression phenotype

Cse4 interactions with other kinetochore proteins are mediated by the N-terminal tail, which is sufficient to recruit components of CCAN (Fischböck-Halwachs *et al*. 2019). The Biggins lab proposed that ectopic localization of Cse4 may titrate centromeric kinetochore proteins (Collins *et al*. 2007). This was confirmed by the Basrai lab and shown to depend on the N-terminal tail (Ciftci-Yilmaz *et al*. 2018 Genetics). Whether this results in formation of functional ectopic kinetochores in budding yeast is unclear. Nevertheless, the titration model suggests that native kinetochore function is compromised by the titration of centromere-bound (or to-be-bound) kinetochore subunits.

The metaphase arrest and the kinetochore phenotype we observed using cell cycle and fluorescence microscopy analyses of CENP-A and chimera-overexpressing cells could be explained by the titration model (Figs. 2 and 3; Supplementary Fig. 3). We showed that CENP-A and to a lesser extent chimera overexpression disrupted both inner and outer kinetochore components (Fig. 3; Supplementary Fig. 3). However, CENP-A does not contain END or the extended N-terminal tail and thus should be less able to recruit native yeast kinetochore components. The CENP-A kinetochore phenotype is much stronger than that of the chimera, and CENP-A lethality is rescued by the addition of the Cse4^N-tail^ fragment to full-length CENP-A (see chimera B in Fig. 2a). However, based on the titration model, one might predict that the addition of the Cse4^N-tail^ to CENP-A would exacerbate titration of kinetochore proteins. We suspect that because the Cse4^N-tail^ also provides a platform for tight regulation of protein levels and localization, it may negate the effects of any residual titration of centromere-bound kinetochore proteins. Therefore, we hypothesize that CENP-A directly interferes with centromeric nucleosomes instead of titrating centromeric kinetochore proteins per se. In other words, overexpression of WT CENP-A may result in a grossly unregulated localization, wreaking havoc throughout chromatin, including destabilizing centromeric nucleosomes, and consequently disrupting kinetochore assembly.

Conversely to WT cells, we found that chimera overexpression in histone-humanized cells was lethal (Fig. 2b). Histone-humanized yeast cells exhibit slow nucleosome remodeling and reduced global transcription (Truong and Boeke 2017). We speculate that the chimeric protein may be either stably incorporated or fail to be evicted by humanized nucleosomes. In either case, this could result in a stronger titration of centromere-bound kinetochore proteins and/or exacerbate global transcription and chromatin abnormalities in histone-humanized yeast. This may also explain the toxicity caused by overexpression of mutant CENP-A^W86R^ in the humanized strain (Fig. 2b). Chimera overexpression did affect kinetochore function in WT cells as determined by fluorescence microscopy (Fig. 3; Supplementary Fig. 3). Moreover, our genetic screen identified SDL between chimera overexpression and deletion of nonessential kinetochore subunits and chromatin factors that are known to be important for centromere function (discussed further below). Collectively, based on these data, we conclude that the chimera phenotype agrees with a model in which an ectopically localized Cse4^N-tail^ titrates away from centromeric kinetochore proteins, resulting in diminished kinetochore function.

The lack of growth defect resulting from chimera (and CENP-A^W86R^) overexpression in WT cells, and the failure to complement Cse4, could be explained by low expression or reduced transcript or protein stability. However, based on our SDL data and the growth phenotypes in histone-humanized yeast, as well as the kinetochore phenotype, we reason that chimera protein is sufficiently produced, but to exclude this possibility, a direct determination of protein levels would be needed.

The Biggins lab isolated and characterized multiple Cse4 mutants that cause lethality when overexpressed in yeast (Collins *et al*. 2007). The Cse4 missense mutants they classified produced significantly higher levels of mutant protein than WT Cse4 when overexpressed using pGAL1. They showed that none of the mutants displaced endogenous Cse4 from the centromere and found no evidence for the titration of centromeric kinetochore proteins. While they concluded that specific mutants likely disrupted kinetochore function, they did not directly assess kinetochores in these mutants. Although they observed biorientation defects in 1 mutant class, they did not observe the declustered outer kinetochore and diffused inner kinetochore phenotype we observe with CENP-A overexpression. They also concluded that a certain class of mutants did not affect kinetochores or replication but likely affected transcription (Collins *et al*. 2007). We have not determined whether the declustered kinetochore foci resulting from CENP-A overexpression represent functional ectopic kinetochores. However, because excessive protein levels of CenH3 result in its mislocalization at regions with high histone turnover, we suspect that this inhibits stabilization of ectopic kinetochore assembly, similar to conditional dicentric chromosomes, where the ectopic centromere is destabilized using *GAL1* promoter (Hill and Bloom 1989; Cook *et al*. 2021). Based on the work by Collins *et al*. discussed above, we interpret the CENP-A overexpression phenotype as resulting from being resistant to proteolysis and localizing both at euchromatin and centromeres, causing both transcriptional and kinetochore disruptions.

### Functional conservation of centromeric remodelers and CenH3 chromatin incorporation

Our genetic screen identified deletions in genes encoding SWI/SNF family subunits of the RSC, INO80, and SWR1 chromatin remodeling complexes as suppressors of CENP-A overexpression (Fig. 4c and d; Supplementary Table 5). Both our CENP-A suppressor and chimera SDL data sets are in agreement with previous genetic screens of *CSE4* overexpression (Ciftci-Yilmaz *et al*. 2018; Eisenstatt *et al*. 2021), suggesting that budding yeast uses shared mechanisms to regulate native Cse4 and human CENP-A. SWI/SNF-like chromatin remodeling factors are important for centromeric function in yeast (Tsuchiya *et al*. 1998; Hsu *et al*. 2003; Baetz *et al*. 2004; Ogiwara *et al*. 2007; Durand-Dubief *et al*. 2012; Verdaasdonk *et al*. 2012; Choi *et al*. 2017). Both INO80 and SWR1 have largely uncharacterized roles in kinetochore function (Krogan *et al*. 2004; Chambers *et al*. 2012). Hildebrand and Biggins found that deletion of the INO80 complex subunit *NHP10*, but not *swr1Δ*, suppressed *CSE4* overexpression–dependent sensitivity in *psh1Δ* cells (Hildebrand and Biggins 2016). In contrast, we found that the absence of multiple INO80/SWR1c subunits, including the Swr1 protein itself, also suppressed CENP-A overexpression. This suggests that both INO80c and SWR1c promote CENP-A ectopic incorporation, whereas only INO80c facilitates Cse4 misincorporation in budding yeast.

The HIR and CAF-1 histone–chaperone complexes are important for Cse4 nucleosome and kinetochore function (Sharp *et al*. 2002; Da Rosa *et al*. 2011; Gkikopoulos *et al*. 2011; Durand-Dubief *et al*. 2012; Deyter and Biggins 2014; Ciftci-Yilmaz *et al*. 2018). HIR and CAF-1 in budding yeast, and HIRA, ATRX, and DAXX in humans, have also been shown to promote deposition of CenH3 into euchromatin (Lacoste *et al*. 2014; Athwal *et al*. 2015; Hewawasam *et al*. 2018; Nye *et al*. 2018). Consistent with Hewawasam *et al*., we found that deletion of the CAF-1 complex subunit *CAC2* suppressed CENP-A overexpression (Supplementary Fig. 2c). Moreover, we found that deletion of HIR subunits, *HIR2*, *HIR3*, and *HPC2*, sensitized chimera-overexpressing cells (Fig. 4f; Supplementary Fig. 4d), indicating enhanced kinetochore dysfunction. In agreement with our results, a recent study that used a high-throughput RNAi screen in human cells to identify factors that prevent CENP-A mislocalization identified both CAF-1 and HIRA subunits and concluded that they prevent chromosomal instability in CENP-A-overexpressing cells (Shrestha *et al*. 2023).

The evolutionarily conserved Yta7^ATAD2/ANCCA^, an ATP-dependent nucleosome segregase, is a Cse4 co-chaperone that targets the Cse4-H4 tetramer to hand it over to the Scm3 chaperone for its deposition at centromeres (Shahnejat-Bushehri and Ehrenhofer-Murray 2020). Notably, we identified deletion of *YTA7* in our genome-wide chimera overexpression screen (Fig. 4f) and found that *yta7Δ* resulted in a strong SDL with chimera overexpression, whereas the negative growth effects with *CSE4* overexpression were considerably milder (Supplementary Fig. 4d). This result agrees with a previous genetic screen with *CSE4* overexpression (Ciftci-Yilmaz *et al*. 2018). These data highlight a possible conserved role for the ATAD2/ANCCA in regulating CENP-A in human cells. Taken together, our data support the notion that CenH3 incorporation is controlled by evolutionarily conserved mechanisms and, thus, that epigenetic regulation of the budding yeast point centromere may be more similar to regional centromeres than is commonly appreciated.

### NDC80c incompatibility

Contrary to previously published work, our data show that partial or complete replacement of the NDC80 outer kinetochore complex by human orthologs is unattainable using our methods. Recently, a new CRISPR-based yeast humanization method, developed by Kachroo and coworkers, was used to humanize most of the α-proteasome (Abdullah *et al*. 2023); thus, it is possible that other approaches may be capable of successfully humanizing the NDC8°c. While we used endogenous promoters to express human Ndc80 subunits as in the previous complementation study (Zheng *et al*. 1999), we did not evaluate whether they were sufficiently produced. However, our result agrees with large-scale complementation studies that also failed to replace NDC80 with the human ortholog (Hamza *et al*. 2015; Kachroo *et al*. 2015). Thus, we suspect that the vast evolutionary divergence between yeast and human kinetochores and the low protein sequence identity may preclude the humanization of kinetochore complexes despite many structural similarities.

The NDC80c is an elongated heterotetrameric structure and interacts with the MIND^MIS12^ subcomplex of the KMN network, Cnn1^CENP-T^ subunit of CCAN, and the DAM1/DASH complex (Wei *et al*. 2005; Ciferri *et al*. 2008; Zahm *et al*. 2023). The protein domains required for interacting with other kinetochore components are not well conserved in humans (Valverde *et al*. 2016; Jenni and Harrison 2018; Zahm *et al*. 2023). Hence, it is unclear to us how the human Ndc80 protein was able to fully complement yeast Ndc80 as previously reported, without any phenotype (Zheng *et al*. 1999). Moreover, yeast and human Ndc80 share only ∼35% identity at the N-terminus (Wigge *et al*. 1998; Zheng *et al*. 1999), the most conserved region, which interacts with microtubules. We would at least expect growth and/or mitotic phenotypes resulting from the complementation. It should be noted that in the reported study, the authors used episomal complementation by human NDC80, and the experiment was performed in a strain in which the first 549-aa sequence of the genomic yeast *NDC80* was replaced by the *URA3* marker, thus retaining *NDC80*'s C-terminal 143-aa coding region. Perhaps this either functions sufficiently along with human Ndc80 for complementation or results in a recombined chimeric protein that can functionally replace yeast Ndc80. In addition, it is possible that a second plasmid variant may have formed and retained the native yeast *NDC80* sequence, a situation we commonly encounter but minimize using the measures described earlier. However, the authors also report that they chromosomally replaced the native genomic *NDC80* with the human ortholog. Further investigation is needed to reconcile these discrepancies.

## Supplementary Material

jkad260_Supplementary_Data

## Data Availability

Strains and plasmids are available upon request. The authors affirm that all data necessary for confirming the conclusions of the article are present within the article, figures, and tables. Supplemental material available at G3 online.
