# Supplementary material for: Humanization reveals pervasive incompatibility of yeast and human kinetochore components
Source: G3 (Bethesda). 2023 Nov 14;14(1):jkad260. doi: 10.1093/g3journal/jkad260 (PMC10755175; doi:10.1093/g3journal/jkad260)
Supplement: jkad260_Supplementary_Data [file jkad260_supplementary_data.zip › Supplemental_Figure_Legends_G3-2023-404663.docx]

**Supplemental figure legends**

**Figure S1. Humanization assay data and analysis of CEN nucleosome humanization candidates.**

1. Schematic of the plasmids used for the CEN nucleosome humanization assays. The Superloser plasmid contains the yeast core histone sequences and recoded *CSE4*. The humanization plasmid contains the human core histone sequences and CENP-A or chimeras.
2. PCR genotyping of humanized candidates was performed on isolated clones that formed colonies after at least seven days. The presence of human histones and CENP-A were verified using primer pairs within H3.1, H4, and H2B, and forward primer in H2A and reverse primer in CENP-A, producing bands ranging from ~150bp to ~600bp. The presence of chimeras was verified using specific primer pairs depending on the chimera in the *yHs∆ dad1^E50D^CSE4+* shuffle strain, and an additional primer pair was used for all chimeras in the *yHs∆ cse4∆* and *yHs∆ dad1^E50D^cse4∆* shuffle strains; the forward primer in the N terminus and the reverse primer in the *CSE4* terminator. We also assessed the presence of the recoded *CSE4* using internal primer pairs that are specific to *rcCSE4.*
3. Example of PCR verification of histone-humanization of *yHs∆ dad1^E50D^cse4∆* and *CSE4+* strains. The presence of *rcCSE4* was only detected in cells that did not have genomic *CSE4* or the positive control (hHs + *CSE4*), suggesting that *rcCSE4* recombination or spontaneous *ura3* mutants. Human H2B was detected in every case.
4. A bar graph displaying the histone humanization data of *yHs∆dad1^E50D^CSE4+* (the same data are presented in Figure 1E. Each black dot represents a single experiment that contained PCR verified humanized colonies after 20 days; cases where no humanization was detected are not plotted (see Table S3 for raw humanization frequency data and for a summary of the PCR genotyping). The red dotted line signifies the level of humanization frequency of the hHs *+ CSE4* positive control.
5. Example of PCR verification of successful and failed humanization of *yHs∆dad1^E50D^CSE4+* shuffle strain. We occasionally observed, such as in this example, that histone-humanized clones had lost the chimera (or CENP-A in other experiments), suggesting plasmid recombination as illustrated, where the *rcCSE4* had replaced the episomal chimera (also observed with CENP-A). The insert shows an image of the humanization plate with large colonies that formed in ~3 days and smaller colonies that formed after 7-14 days (the image shown was captured after 20 days). Green arrows indicate successfully humanized colonies that had retained the chimera and lost *rcCSE4* (but contain the genomic *CSE4*), red arrows indicate colonies that failed to humanize with chimeras, that is, had lost the chimera and retained *rcCSE4*, but successfully humanized the core histones.
6. Example of PCR verification of failed CenH3 nucleosome humanization in *yHs∆ dad1^E50D^cse4∆* shuffle strain. Occasionally, we observed colonies forming after 7-14 days, similar to histone humanization in the *dad1^E50D^* background. However, in every case tested, PCR genotyping revealed that the clones contained hHs and *rcCSE4* and often lost the chimera as in this example, and CENP-A in other experiments*,* similar to the example in panel E. Images of plates from an experiment with two replicates shows that the histone humanized colonies, that were PCR genotyped and had retained *rcCSE4* and lost the chimera, formed colonies after 7 days (the PCR genotyping was done after 20 days of growth).
7. Multiple amino acid sequence alignment of the histone-fold domains of CenH3 from *S. cerevisiae* and *H. sapiens*, and other relevant species discussed in the main text; *N. gla = Nakaseomyces glabratus; P. ang = Pichia angusta; P. fal = Plasmodium falciparum.*
8. Plasmid-shuffle assay with indicated pGAL1 plasmids in cse4∆ strain containing CSE4 on a URA3 plasmid. Log-phase cultures of were diluted to the same OD600 level and ten-fold serial dilutions were spotted onto synthetic media without and containing 5-FOA. The image shown was captured after 3 days of growth at 30˚C, but we did keep the plates for 40 days at 30˚C in case we would find complemented colonies.
9. Ten-fold serial dilution spot assay with indicated plasmids in the stable histone-humanized *dad1^E50D^* strain on media containing either 2% dextrose (expression off), or 2% galactose (expression on). The images were captured after 7 days of growth at 30˚C.

**Figure S2. The effects of deleting PSH1, NHP10, and CAC2 on CENP-A and chimera overexpression.**

1. Spot-assay with the indicated plasmids in wild-type and *psh1∆* cells. Log-phase cultures were diluted to the same OD_600_ level and ten-fold serial dilutions were prepared and spotted onto synthetic media containing 2% dextrose (expression off), 2% galactose and 1% raffinose (high expression), and 0.1% galactose and 2% raffinose (lower expression).
2. Spot-assay with the indicated plasmids in wild-type and *psh1∆* cells. Log-phase cultures were diluted to the same OD_600_ level and ten-fold serial dilutions were prepared and spotted onto synthetic media containing 2% dextrose (expression off) and 2% galactose and 1% raffinose (expression on).
3. A spot-assay with the indicated plasmids in wild-type, *nhp10∆,* and *cac2∆* cells. Log-phase cultures were diluted to the same OD_600_ level and ten-fold serial dilutions were prepared and spotted onto synthetic media containing 2% dextrose (expression off), 2% galactose and 1% raffinose (high expression), and 0.1% galactose and 2% raffinose (lower expression).

**Figure S3. Overexpression of CENP-A disrupts Mtw1 and Cep3 foci**

1. The kinetochore foci of the Mtw1-GFP cells containing empty vector (n=124), pGAL1-CENP-A^W86R^ (n=102), pGAL1-CENP-A (n=71), and pGAL1-chimera C (n=103) plasmids were analyzed using fluorescence microscopy after galactose induction for five hours and abnormal diffused or declustered Cep3-GFP foci were quantified. Fishers exact statistical test; p-values * = p < 0.05; **** = p < 10^-5^. Error bars indicate 95% binomial CI. Representative micrographs are shown on the right. Scale bars indicate 5 µm.
2. The kinetochore foci of the Cep3-GFP cells containing empty vector (n=56), pGAL1-CENP-A^W86R^ (n=52), pGAL1-CENP-A (n=30), and pGAL1-chimera C (n=36) plasmids were analyzed using fluorescence microscopy after galactose induction for five hours and abnormal declustered Mtw1-GFP foci were quantified. Fishers exact statistical test; p-values **** = p < 10^-5^. Error bars indicate 95% binomial CI. Representative micrographs are shown on the right. White arrows highlight the Cep3-GFP phenotype observed (see main text for details). Scale bars indicate 5 µm.
3. Violin plot showing fluorescence intensities of Cep3-GFP kinetochore foci in cells containing empty vector (n=103), pGAL1-CENP-A^W86R^ (n=57), pGAL1-CENP-A (n=91), pGAL1-chimera C (n=64) plasmids. Culture conditions are the same as those in panel B. Statistical significance was evaluated using unpaired two-tailed student’s t-test; p-value ** = p < 0.005. Cells containing no detectable or very diffused Cep3-GFP signals were excluded from the analysis.

**Figure S4. Validation of genetic interactions of CENP-A and chimera overexpressions**

1. Spot assay of wild-type and indicated INO80/SWR1 deletion strains identified as CENP-A-overexpression suppressors in the genome-wide screen, except for *nhp10∆*, which we included for comparison purposes, since we found that it suppressed CENP-A overexpression in Figure S2 C. Log-phase cultures of cells containing the indicated plasmids were diluted to the same OD_600_ level and ten-fold serial dilutions were prepared and spotted onto synthetic media containing 2% dextrose (not shown) and 2% galactose and 1% raffinose.
2. Spot assay of wild-type and indicated RSC deletion strains identified as CENP-A-overexpression suppressors in the genome-wide screen. Log-phase cultures of cells containing the indicated plasmids were diluted to the same OD_600_ level and ten-fold serial dilutions were prepared and spotted onto synthetic media containing 2% dextrose (not shown) and 2% galactose and 1% raffinose.
3. Spot assay of wild-type and indicated kinetochore deletion strains identified as negative interactors of chimera C overexpression in the genome-wide screen. Log-phase cultures of cells containing the indicated plasmids were diluted to the same OD_600_ level and ten-fold serial dilutions were prepared and spotted onto synthetic media containing 2% dextrose and 2% galactose and 1% raffinose.
4. Spot assay of *yta7∆* and indicated HIR complex deletion strains identified as negative interactors of chimera C overexpression in the genome-wide screen. Log-phase cultures of cells containing the indicated plasmids were diluted to the same OD_600_ level and ten-fold serial dilutions were prepared and spotted onto synthetic media containing 2% dextrose and 2% galactose and 1% raffinose.

**Figure S5. NDC80 complex subunit complementation analysis**

1. Overviews of three strategies for humanizing the NDC80 complex. 1) We used the dual-plasmid shuffle assay to complement deletions made by CRISPR/Cas9 of NDC80c subunits (see main text for details). 2) Instead of deleting the NDC80c subunits from the genome as in strategy 1, we used the hNDC80c counterpart sequences flanked by *S. cerevisiae* promoters and terminators as repair templates when cutting the genomic endogenous genes using CRISPR/Cas9 and the Superloser plasmid containing the yNDC80c subunits was used for ensuring viability. 3) As in strategy 2, we used CRISPR/Cas9 to cut the endogenous genomic yNDC80c subunit sequences separately and repaired using the human sequences, but we did not include the Superloser plasmid for viability.
2. Example complementation spot assay of complete genomic replacement of yeast NDC80c subunits by human orthologs. Complementation experiments were also performed by plating various amounts of cells (see Table S6).
3. Example complementation spot assay of individual genomic replacement of yeast *NDC80* and *SPC25* by human orthologs. Complementation experiments were also performed by plating various amounts of cells (see Table S6).
4. Example complementation spot assay of double and triple genomic replacement of indicated yeast NDC80c subunits by human orthologs. Complementation experiments were also performed by plating various amounts of cells (see Table S6). Note that the colonies on the plates containing 5-FOA appeared after 3 days and were PCR validated for humanization, but in all cases contained the yeast NDC80c subunits.
5. PCR genotyping primer pairs were designed to amplify ~150-250 bp internal products from each yeast native, yeast recoded, and human NDC80c subunit sequence.
6. Example of PCR genotyping of clones that formed colonies on 5-FOA containing media. This example shows that individual *NDC80* complementation failed and the clones retained both the recoded yeast *NDC80* and the human ortholog.
7. Example of PCR genotyping of candidate NDC80 humanized clones containing genomic hNDC80c that formed colonies (total 8 colonies) after 10 days. In every case the clones retained the recoded yeast NDC80c sequences.
