# Supplementary material for: Humanization reveals pervasive incompatibility of yeast and human kinetochore components
Source: G3 (Bethesda). 2023 Nov 14;14(1):jkad260. doi: 10.1093/g3journal/jkad260 (PMC10755175; doi:10.1093/g3journal/jkad260)
Supplement: jkad260_Supplementary_Data [file jkad260_supplementary_data.zip › Table_S1_G3-2023-404663.pdf]

Strain name	MAT	*Genotype*	Plasmid	Markers	Source
yGOL001	α	*his3Δ200 leu2Δ0 lys2Δ0 trp1Δ63 ura3Δ0 met15Δ0 hta2-htb2Δ0 hta1-htb1Δ0 hht1-hhf1Δ0 hht2-hhf2Δ0 dad1-E50D*	pDT139-CSE4	TRP	This study
yGOL014	α	*his3Δ200 leu2Δ0 lys2Δ0 trp1Δ63 ura3Δ0 met15Δ0 hta2-htb2Δ0 hta1-htb1Δ0 hht1-hhf1Δ0 hht2-hhf2Δ0 dad1-E50D cse4Δ::HIS5MX6*	pDT139-CSE4	TRP/HIS	This study
yGOL067	α	*his3Δ200 leu2Δ0 lys2Δ0 trp1Δ63 ura3Δ0 met15Δ0 hta2-htb2Δ0 hta1-htb1Δ0 hht1-hhf1Δ0 hht2-hhf2Δ0 dad1-E50D*	pGOL011	URA	This study
yGOL069	α	*his3Δ200 leu2Δ0 lys2Δ0 trp1Δ63 ura3Δ0 met15Δ0 hta2-htb2Δ0 hta1-htb1Δ0 hht1-hhf1Δ0 hht2-hhf2Δ0 dad1-E50D cse4Δ::HIS5MX6*	pGOL011	URA	This study
yGOL091-095	a	*leu2Δ0 met15Δ0 ura3Δ0 his3Δ1 ndc80Δ*	pGOL87	URA	This study
yGOL097	a	*leu2Δ0 met15Δ0 ura3Δ0 his3Δ1 ndc80Δ::hNDC80*	pGOL87	URA	This study
yGOL101-102	a	*leu2Δ0 met15Δ0 ura3Δ0 his3Δ1 spc25Δ*	pGOL87	URA	This study
yGOL104-105	a	*leu2Δ0 met15Δ0 ura3Δ0 his3Δ1 spc25Δ::hSPC25*	pGOL87	URA	This study
yGOL107	a	*leu2Δ0 met15Δ0 ura3Δ0 his3Δ1 ndc80Δ spc25Δ*	pGOL87	URA	This study
yGOL115-116	a	*leu2Δ0 met15Δ0 ura3Δ0 his3Δ1 spc24Δ*	pGOL87	URA	This study
yGOL118-119	a	*leu2Δ0 met15Δ0 ura3Δ0 his3Δ1 nuf2Δ*	pGOL87	URA	This study
yGOL121	a	*leu2Δ0 met15Δ0 ura3Δ0 his3Δ1 ndc80Δ::hNDC80 spc25::hSPC25*	pGOL87	URA	This study
yGOL129	a	*leu2Δ0 met15Δ0 ura3Δ0 his3Δ1 ndc80Δ spc25Δ spc24Δ*	pGOL87	URA	This study
yGOL135	a	*leu2Δ0 met15Δ0 ura3Δ0 his3Δ1 ndc80Δ spc25Δ nuf2Δ*	pGOL87	URA	This study
yGOL141	a	*leu2Δ0 met15Δ0 ura3Δ0 his3Δ1 ndc80Δ spc25Δ spc24::hSPC24*	pGOL87	URA	This study
yGOL144	a	*leu2Δ0 met15Δ0 ura3Δ0 his3Δ1 ndc80Δ spc25Δ nuf2::hNUF2*	pGOL87	URA	This study
yGOL148-152	a	*leu2Δ0 met15Δ0 ura3Δ0 his3Δ1 ndc80Δ::hNDC80 spc25::hSPC25 spc24::hSPC24*	pGOL87	URA	This study
yGOL153-160	a	*leu2Δ0 met15Δ0 ura3Δ0 his3Δ1 ndc80Δ spc25Δ spc24Δ nuf2Δ*	pGOL87	URA	This study
yGOL162-166	a	*leu2Δ0 met15Δ0 ura3Δ0 his3Δ1 ndc80Δ::hNDC80 spc25::hSPC25 spc24::hSPC24 nuf2::hNUF2*	pGOL87	URA	This study
yGOL168	a	*leu2Δ0 met15Δ0 ura3Δ0 his3Δ1 spc24Δ ndc80Δ*	pGOL87	URA	This study
yGOL171	a	*leu2Δ0 met15Δ0 ura3Δ0 his3Δ1 spc24Δ spc25Δ*	pGOL87	URA	This study
yGOL174	a	*leu2Δ0 met15Δ0 ura3Δ0 his3Δ1 nuf2Δ ndc80Δ*	pGOL87	URA	This study
yGOL177	a	*leu2Δ0 met15Δ0 ura3Δ0 his3Δ1 nuf2Δ spc25Δ*	pGOL87	URA	This study
yGOL180	a	*leu2Δ0 met15Δ0 ura3Δ0 his3Δ1 nuf2Δ spc24Δ*	pGOL87	URA	This study
yGOL187	α	*his3Δ200 leu2Δ0 lys2Δ0 trp1Δ63 ura3Δ0 met15Δ0 hta2-htb2Δ0 hta1-htb1Δ0 hht1-hhf1Δ0 hht2-hhf2Δ0 cse4Δ::HIS5MX6*	pGOL011	URA/HIS	This study
yGOL188	a	*leu2Δ0 met15Δ0 ura3Δ0 his3Δ1 cse4Δ::HIS5MX6 [GAL1p-CSE4-CYC1t]*	pGOL018	URA/HIS	This study
GFP strains	a	*leu2Δ0 met15Δ0 ura3Δ0 his3Δ1 ORF-GFP::HIS5MX6*		HIS	Huh et al. 2003
Deletion strains	a	*his3Δ1 leu2Δ0 met15Δ0 ura3Δ0 orfΔ::KANMX*		KAN	Winzeler et al. 1999
yDT67	α	*his3Δ200 leu2Δ0 lys2Δ0 trp1Δ63 ura3Δ0 met15Δ0 hta2-htb2Δ0 hta1-htb1Δ0 hht1-hhf1Δ0 hht2-hhf2Δ0*	pDT105	TRP	Truong and Boeke 2017
yDT180	α	*his3Δ200 leu2Δ0 lys2Δ0 trp1Δ63 ura3Δ0 met15Δ0 hta2-htb2Δ0 hta1-htb1Δ0 hht1-hhf1Δ0 hht2-hhf2Δ0 dad1-E50D histone humanized*	pDT109	TRP	Truong and Boeke 2017
BY4741	a	*his3Δ1 leu2Δ0 met15Δ0 ura3Δ0*			Brachmann et al. 1998
W8164-2B	α	*Universal Donor Strain (UDS) CEN1-16::Gal-Kl-URA3 (derivative of W303 can1-100 his3-11,15 leu2-3,112 ura3-1 RAD5)*		URA	Reid et al. 2011

yMAH700	α	*his3Δ200 leu2Δ0 lys2Δ0 trp1Δ63 ura3Δ0 met15Δ0 hta2-htb2Δ0 hta1-htb1Δ0 hht1-hhf1Δ0 hht2-hhf2Δ0 dad1-E50D*	pDT139	URA	Haase et al. 2023
