# Supplementary material for: Humanization reveals pervasive incompatibility of yeast and human kinetochore components
Source: G3 (Bethesda). 2023 Nov 14;14(1):jkad260. doi: 10.1093/g3journal/jkad260 (PMC10755175; doi:10.1093/g3journal/jkad260)
Supplement: jkad260_Supplementary_Data [file jkad260_supplementary_data.zip › Table_S2_G3-2023-404663.pdf]

Plasmid name	Alternate name	Markers	Description	Source
pGOL001	hHist.&Chimera A	AMP/TRP	pRS414-hH3.1_hH4_hH2A_hH2B_Cse4(N61)-CENPA(NΔ9)chimera (S.cer promoters & terminators = TEs)	This study
pGOL005	hHist.&Chimera B	AMP/TRP	pRS414-hH3.1_hH4_hH2A_hH2B_Cse4(N61)-AA-CENPA(NΔ1)chimera (S.cer TEs)	This study
pGOL006	Superloser V3	KAN/URA	pDT139-HTA2-HTB2-HHT1-HHF1/URA3/URA3syn/GALCEN (same as pDT139 except cloning site was replaced with PvuII/KasI/SfoI sites)	This study
pGOL011	yHs+rcCSE4 Superloser	KAN/URA	pGOL006-HTA2-HTB2-HHT1-HHF1-recodedCSE4/URA3/URA3syn/GALCEN	This study
pGOL012	hHs+Chimera C	AMP/TRP	pRS414-hH3.1_hH4_hH2A_hH2B_Cse4(N135)-CENPA(HFD)chimera (S.cer TEs)	This study
pGOL017	pGAL-CENPA	AMP/LEU	pAV115-GAL1p-CENPA-CYC1t	This study
pGOL018	pGAL-CSE4_URA3	AMP/URA	pAV116-GAL1p-CSE4-CYC1t	This study
pGOL025	pGAL-CSE4_LEU2	AMP/LEU	pAV115-GAL1p-CSE4-CYC1t	This study
pGOL026	pGAL-Chimera A	AMP/LEU	pAV115-GAL1p-Cse4(N61)-AA-CENPA(NΔ1)chimera-CYC1t	This study
pGOL027	pGAL-Chimera B	AMP/LEU	pAV115-GAL1p-Cse4(N61)-AA-CENPA(NΔ1)chimera-CYC1t	This study
pGOL028	pGAL-Chimera C	AMP/LEU	pAV115-GAL1p-Cse4(N135)-CENPA(HFD)chimera-CYC1t	This study
pGOL031	pGAL-CENP-A-W86R	AMP/LEU	pAV115-GAL1p-CENP-A-W86R-CYC1t	This study
pGOL032	pGAL-Chimera D	AMP/LEU	pAV115-GAL1p-Cse4(N135)-CENPA(HFD-yL1)chimera-CYC1t	This study
pGOL049	hHs+Chimera D	AMP/TRP	pRS414-hH3.1_hH4_hH2A_hH2B_Cse4(N135)-CENPA(HFD-yL1)chimera (S.cer TEs)	This study
pGOL052	hHs+Chimera E	AMP/TRP	pRS414-hH3.1_hH4_hH2A_hH2B_Cse4(N135,HFD1)-CENPA(HFD2,3)chimera (S.cer TEs)	This study
pGOL053	hHs+Chimera F	AMP/TRP	pRS414-hH3.1_hH4_hH2A_hH2B_Cse4(N135,HFD2)-CENPA(HFD1,3)chimera (S.cer TEs)	This study
pGOL054	hHs+Chimera G	AMP/TRP	pRS414-hH3.1_hH4_hH2A_hH2B_Cse4(N135,HFD3)-CENPA(HFD1,2)chimera (S.cer TEs)	This study
pGOL055	hHs+Chimera H	AMP/TRP	pRS414-hH3.1_hH4_hH2A_hH2B_Cse4(N135,HFD1,2)-CENPA(HFD3)chimera (S.cer TEs)	This study
pGOL056	hHs+Chimera I	AMP/TRP	pRS414-hH3.1_hH4_hH2A_hH2B_Cse4(N135,HFD1,3)-CENPA(HFD2)chimera (S.cer TEs)	This study
pGOL057	hHs+Chimera J	AMP/TRP	pRS414-hH3.1_hH4_hH2A_hH2B_Cse4(N135,HFD2,3)-CENPA(HFD1)chimera (S.cer TEs)	This study
pGOL066	hHs+wtCSE4	AMP/TRP	pRS414-hH3.1_hH4_hH2A_hH2B_CSE4 (S.cer TEs)	This study
pGOL081	hNDC80c plasmid	AMP/LEU	pRS415-hNDC80-hNUF2-hSPC24-hSPC25_S.cer Tes (sequenced)	This study
pGOL084	hHs_hH2B-K117R + Chimera C	AMP/TRP	pRS414-hH3.1_hH4_hH2A_hH2B-K117R_Cse4(N135)-CENPA(HFD)chimera (S.cer TEs)	This study
pGOL085	hHs+Chimera K	AMP/TRP	pRS414-hH3.1_hH4_hH2A_hH2B_Cse4(N135)-CENPA(yL1+4K-swap-back)chimera (S.cer TEs)	This study
pGOL086	hHs+Chimera L	AMP/TRP	pRS414-hH3.1_hH4_hH2A_hH2B_Cse4(N135)-CENPA(CATD-swap-back)chimera (S.cer TEs)	This study
pGOL087	yNDC80c Superloser	KAN/URA	pGOL006-recoded_yNDC80-yNUF2-ySPC24-ySPC25/URA3/URA3syn/GALCEN (S.eub TEs)	This study
pGOL093	NDC80 gRNA	AMP/LEU	p425-SNR52p-NotI(NDC80-gRNA)-SUP4t	This study
pGOL095	SPC25 gRNA	AMP/LEU	p425-SNR52p-NotI(SPC25-gRNA)-SUP4t	This study
pGOL097	SPC24 gRNA	AMP/LEU	p425-SNR52p-NotI(SPC24-gRNA)-SUP4t	This study
pGOL099	NUF2 gRNA	AMP/LEU	p425-SNR52p-NotI(NUF2-gRNA)-SUP4t	This study

pGOL104	pRS415-hNDC80	AMP/LEU	pRS415-hNDC80_S.cer TEs	This study
pGOL105	pRS415-hNUF2	AMP/LEU	pRS415-hNUF2_S.cer TEs	This study
pGOL106	pRS415-hSPC24	AMP/LEU	pRS415-hSPC24_S.cer TEs	This study
pGOL107	pRS415-hSPC25	AMP/LEU	pRS415-hSPC25_S.cer TEs	This study
pDT139	yHs Superloser	KAN/URA	HTA2-HTB2-HHT1-HHF1 /URA3/URA3syn/GALCEN	Haase et al. 2019
pDT109	hHs plasmid	AMP/TRP	pRS414 with human core histones (H3.1 H4 H2A H2B) with HHT2F2HTA1B1 PROs/TERs	Truong and Boeke. 2017
pNA525	LEU2 guide RNA expression vector	AMP/LEU	p425-SNR52p-NotI(gRNA)-SUP4t - NotI site for inserting any gRNA using Gibson Assembly.	DiCarlo et al. 2013
pNA0519	Cas9 vector	AMP/HIS	pRS413-TEF1p-Cas9-CYC1t - Cas9 fragment cloned into pRS413	DiCarlo et al. 2013
pAV115	empty LEU2 plasmid	AMP/LEU	pRS415-yGG_acceptor vector	Agmon et al. 2015

Haase *et al.* ; https://doi.org/10.1534/g3.119.400325

Truong and Boeke.; https://doi.org/10.1016/j.cell.2017.10.043

Brachmann et al.; https://doi.org/10.1002/(SICI)1097-0061(19980130)14:2<115::AID-YEA204>3.0.CO;2-2

Agmon et al.; https://doi.org/10.1021/sb500372z

DiCarlo et al.; https://doi.org/10.1093/nar/gkt135
