# Supplementary figures and images for: Humanization reveals pervasive incompatibility of yeast and human kinetochore components
Source: G3 (Bethesda). 2023 Nov 14;14(1):jkad260. doi: 10.1093/g3journal/jkad260 (PMC10755175; doi:10.1093/g3journal/jkad260)

**Figure S1. Humanization assay data and analysis of CEN nucleosome humanization candidates.**

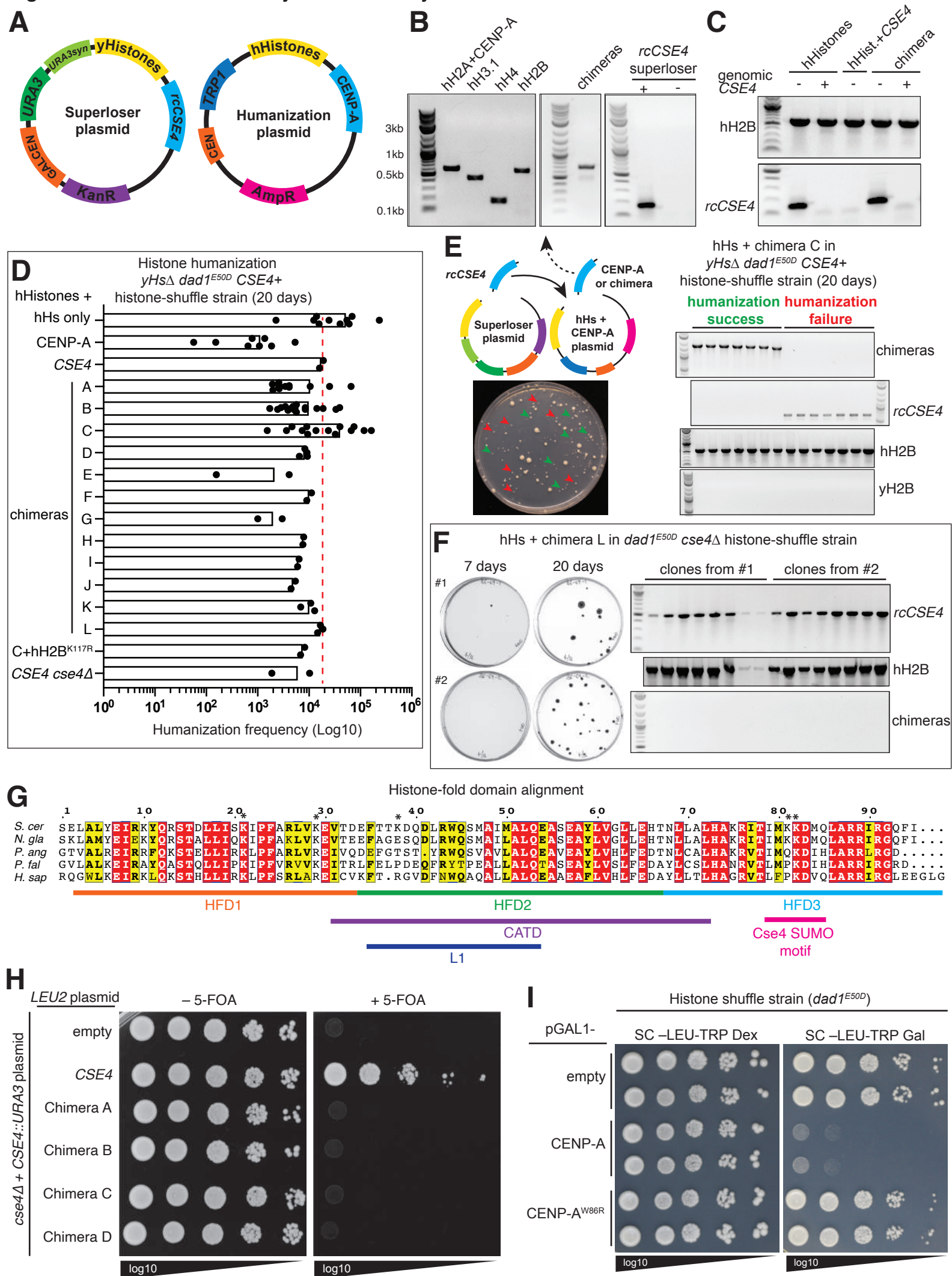

Supplement: jkad260_Supplementary_Data [file jkad260_supplementary_data.zip › Figure_S1_G3-2023-404663.pdf]

**Figure S2. The effects of deleting *PSH1*, *NHP10*, and *CAC2* on CENP-A and chimera overexpression.**

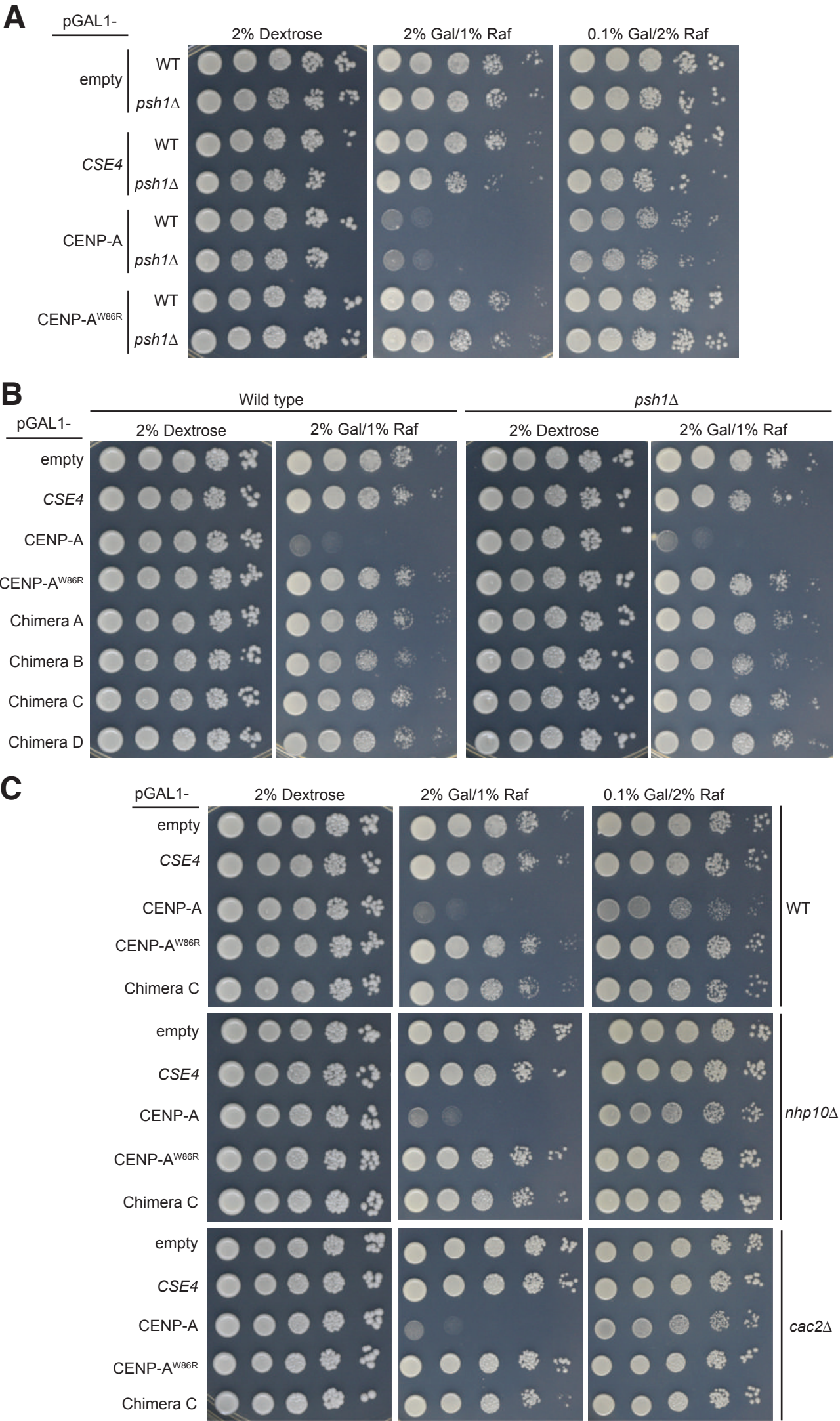

Supplement: jkad260_Supplementary_Data [file jkad260_supplementary_data.zip › Figure_S2_G3-2023-404663.pdf]

**Figure S3. Overexpression of CENP-A disrupts Mtw1 and Cep3 foci**

**A**

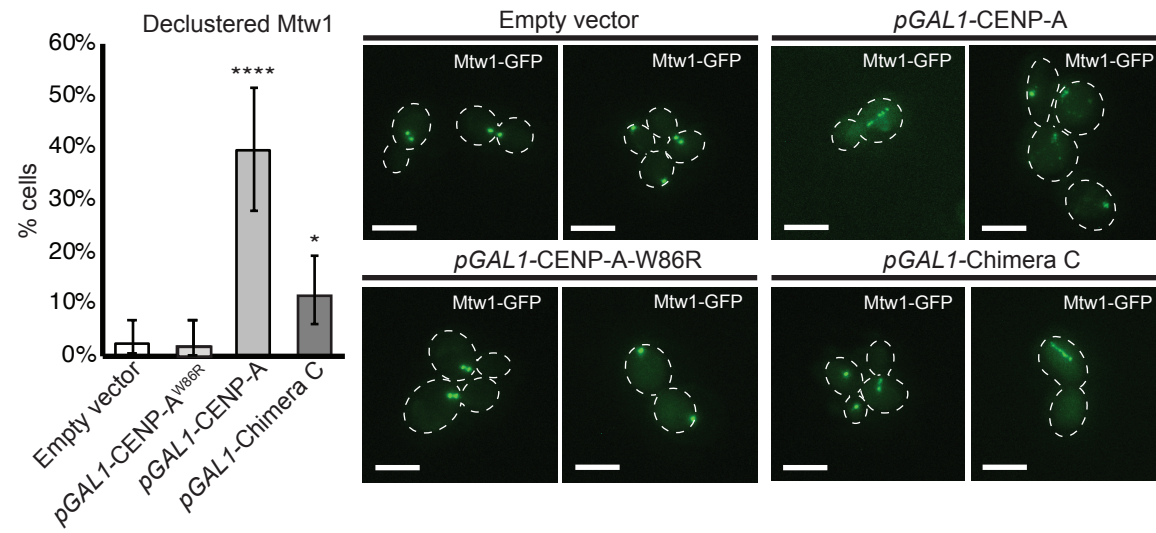

**B**

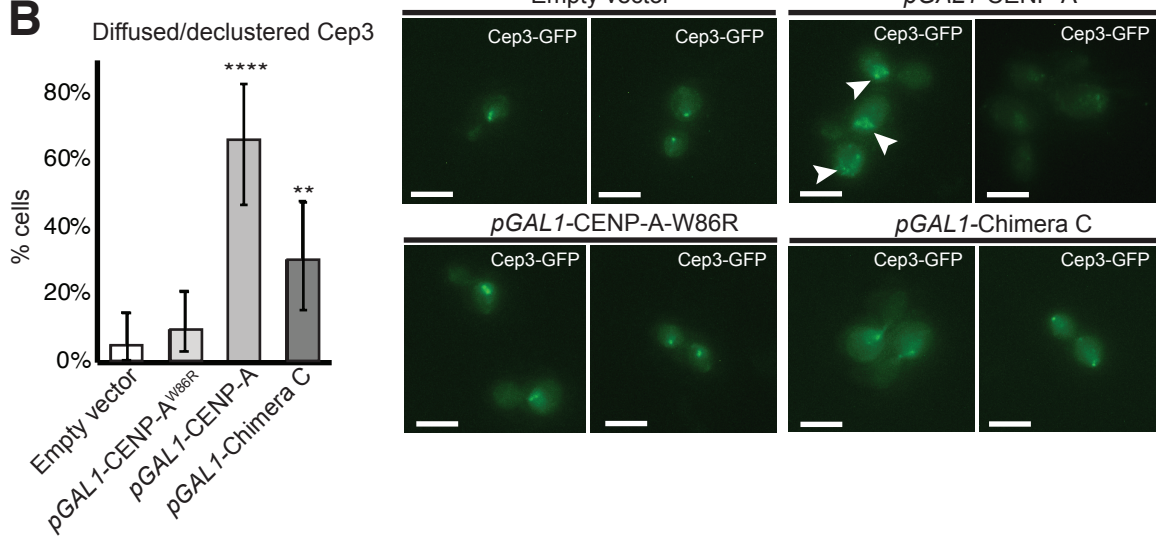

**C**

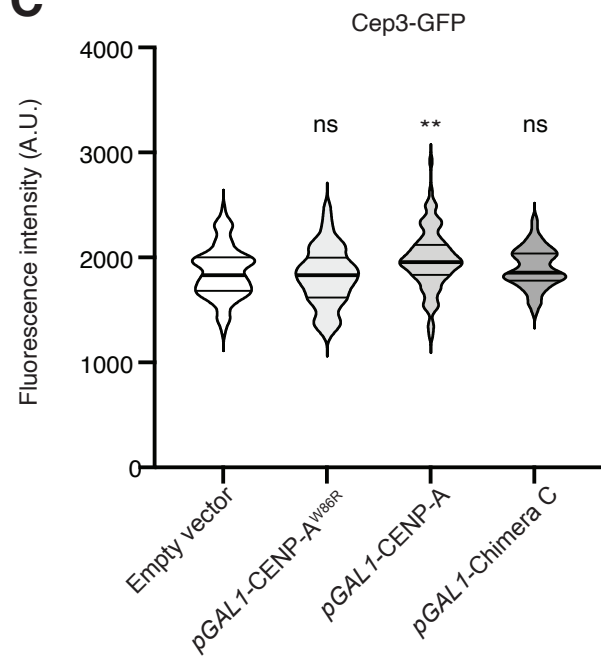

Supplement: jkad260_Supplementary_Data [file jkad260_supplementary_data.zip › Figure_S3_G3-2023-404663.pdf]

**Figure S4. Validation of genetic interactions of CENP-A and chimera overexpressions**

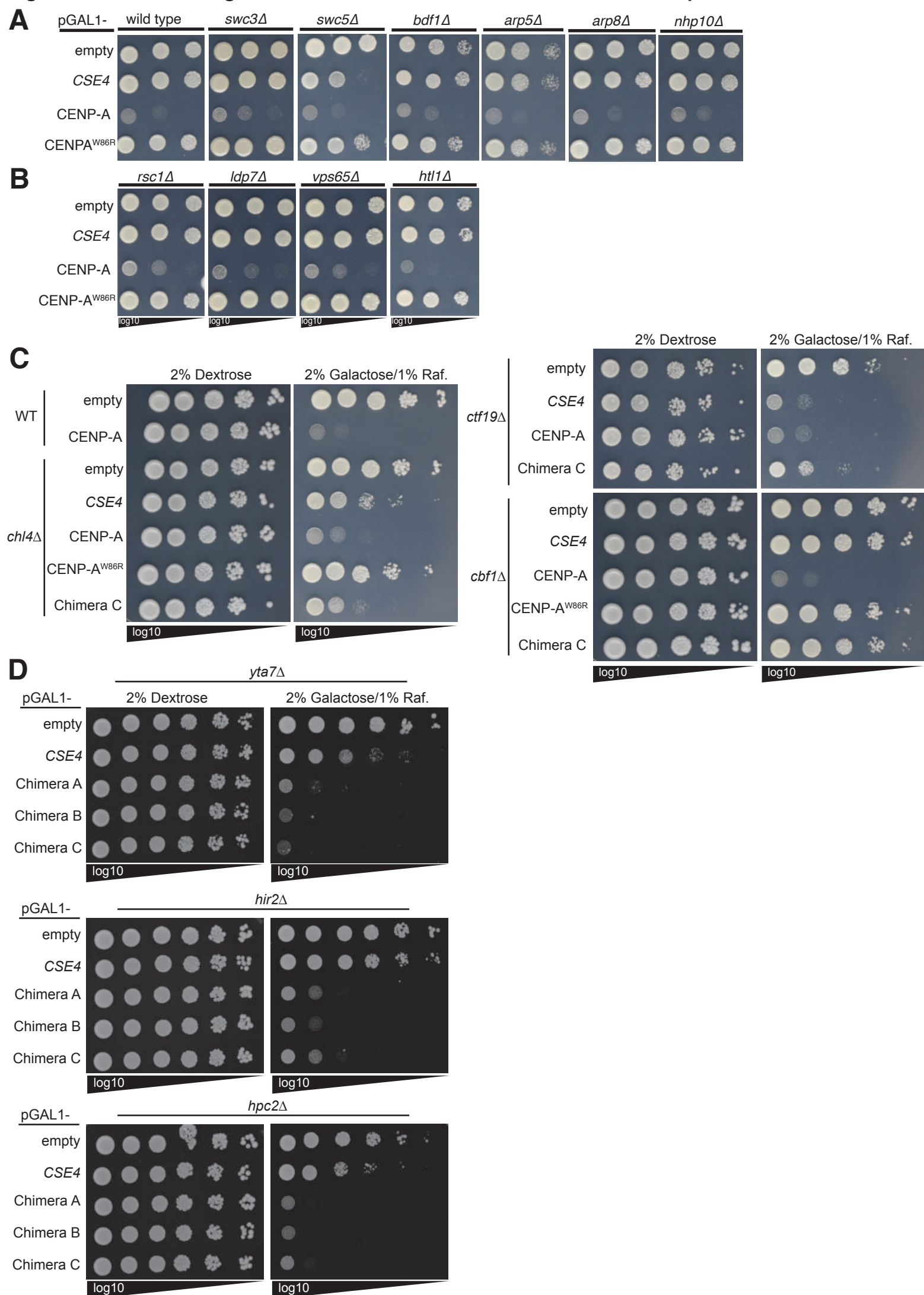

Supplement: jkad260_Supplementary_Data [file jkad260_supplementary_data.zip › Figure_S4_G3-2023-404663.pdf]

Figure S5. NDC80 complex subunit complementation analysis

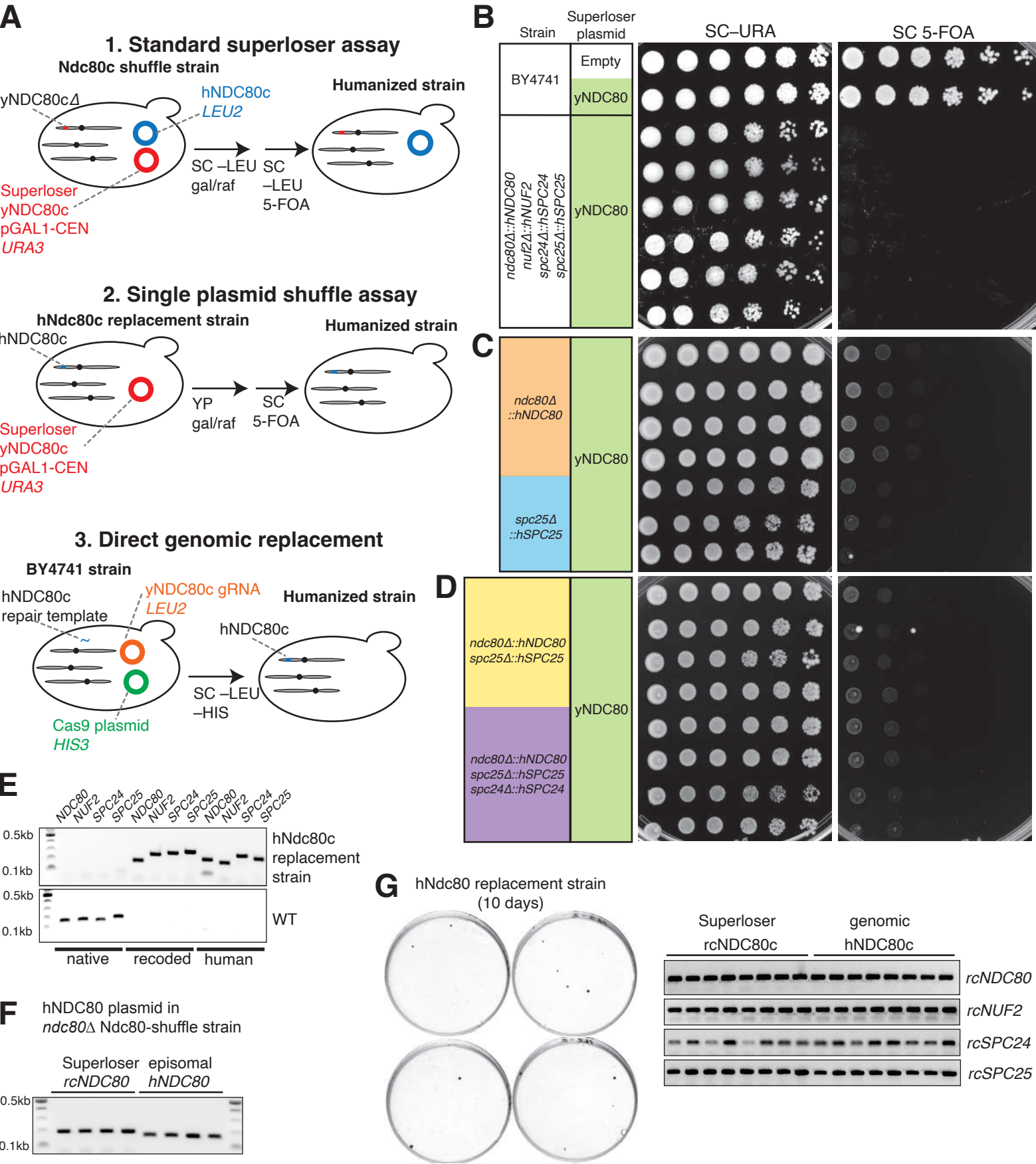

Supplement: jkad260_Supplementary_Data [file jkad260_supplementary_data.zip › Figure_S5_G3-2023-404663.pdf]
